# GnIH secreted by green light exposure, regulates bone mass through the activation of Gpr147

**DOI:** 10.1038/s41413-024-00389-7

**Published:** 2025-01-21

**Authors:** Yu You, Konglin Huo, Liang He, Tongyue Wang, Lei Zhao, Rong Li, Xiaoqing Cheng, Xuebin Ma, Zhiying Yue, Stefan Siwko, Ning Wang, Lujian Liao, Mingyao Liu, Jian Luo

**Affiliations:** 1 Shanghai Key Laboratory of Regulatory Biology, Institute of Biomedical Sciences and School of Life Sciences, East China Normal University, Shanghai, PR China; 2 Yangzhi Rehabilitation Hospital (Shanghai Sunshine Rehabilitation Center), Tongji University School of Medicine, Shanghai, PR China; 3 Precision Research Center for Refractory Diseases, Shanghai General Hospital,Shanghai Jiaotong University, School of Medicine, Shanghai, PR China; 4 Department of Translational Medical Sciences, Institute of Biosciences and Technology, Texas A&M University Health Science Center, Houston, TX 77030 USA; 5 Leicester Cancer Research Centre, Department of Genetics and Genome Biology, University of Leicester, Leicester, UK; 6 Division of Clinical Medicine, School of Medicine and Population Health, University of Sheffield, Sheffield, UK

**Keywords:** Bone, Endocrine system and metabolic diseases

## Abstract

Reproductive hormones associated with the hypothalamic-pituitary-gonadal (HPG) axis are closely linked to bone homeostasis. In this study, we demonstrate that Gonadotropin inhibitory hormone (GnIH, one of the key reproductive hormones upstream of the HPG axis) plays an indispensable role in regulating bone homeostasis and maintaining bone mass. We find that deficiency of GnIH or its receptor Gpr147 leads to a significant reduction in bone mineral density (BMD) in mice primarily by enhancement of osteoclast activation in vivo and in vitro. Mechanistically, GnIH/Gpr147 inhibits osteoclastogenesis by the PI3K/AKT, MAPK, NF-κB and Nfatc1 signaling pathways. Furthermore, GnIH treatment was able to alleviate bone loss in aging, ovariectomy (OVX) or LPS-induced mice. Moreover, the therapy using green light promotes the release of GnIH and rescues OVX-induced bone loss. In humans, serum GnIH increases and bone resorption markers decrease after green light exposure. Therefore, our study elucidates that GnIH plays an important role in maintaining bone homeostasis via modulating osteoclast differentiation and demonstrates the potential of GnIH therapy or green light therapy in preventing osteoporosis.

## Introduction

The homeostasis of bone mass is maintained through a dynamic balance between bone formation and bone resorption.^1–3^ Disruption of either process can result in an imbalance of bone homeostasis, ultimately leading to pathological conditions such as osteoporosis.^4–7^ Hormones associated with the HPG axis include GnIH, kisspeptin, Gonadotropin-releasing hormone (GnRH), Follicle-stimulating hormone (FSH), Luteinizing hormone (LH), estrogen, and testosterone.^8,9^ Evidence suggests that, besides estrogens and androgens, upstream reproductive hormones associated with the HPG axis also play a crucial role in regulating bone homeostasis,^8,10,11^ including FSH and LH,^12^ GnRH,^8,13^ kisspeptin,^14,15^ and upstream key reproductive hormones can directly affect bone tissue. However, treatments targeting these hormones either have minimal or no direct effects on osteoblasts and osteoclast,^8^ or lead to severe side effects loss.^16–21^ Therefore, it is imperative to investigate the regulatory roles of other upstream reproductive hormones in maintaining bone homeostasis and explore their therapeutical potentials of preventing and treating orthopedic diseases.^8^

GnIH is a novel neuropeptide of hypothalamic origin,^22–25^ and it is a key upstream reproductive hormone involved in the regulation of the HPG axis and acts to inhibit reproduction.^26–29^ Accumulating evidence suggests that GnIH acts on GnRH neurons by binding G protein-coupled receptor 147 (GPR147) and regulates the function of GnRH release and synthesis.^30–34^ The use of GnRH agonists in clinical practice has shown to reduce bone mass and increase the risk of fractures. In contrast, the use of GnRH antagonists can significantly decrease the incidence of musculoskeletal events.^35^ These observations suggest that GnIH may play a protective role in bone. However, the molecular events underlying GnIH regulation of bone homeostasis is unclear. Intriguingly, multiple studies have revealed that green light enhances the synthesis and release of GnIH in poultry animals.^36,37^ Green light therapy has demonstrated analgesic effects in both human and murine subjects,^38^ however, it is unknown whether green light therapy provide benefits for other diseases including osteoporosis.

In this study, we provide evidence showing that GnIH participates in regulating bone homeostasis and maintaining bone mass by suppressing the osteoclast differentiation, and that green light therapy promotes the release of GnIH and prevents bone loss by blocking osteoclast genesis in osteoporosis mouse model, as well as downregulates serum levels of bone resorption markers in human subjects. Therefore, our study suggests that green light therapy can promote GnIH release, which may provide a new strategy for preventing and treating osteoporosis.

## Results

### GnIH/Gpr147 signaling regulates bone mass

**Fig. 1:**
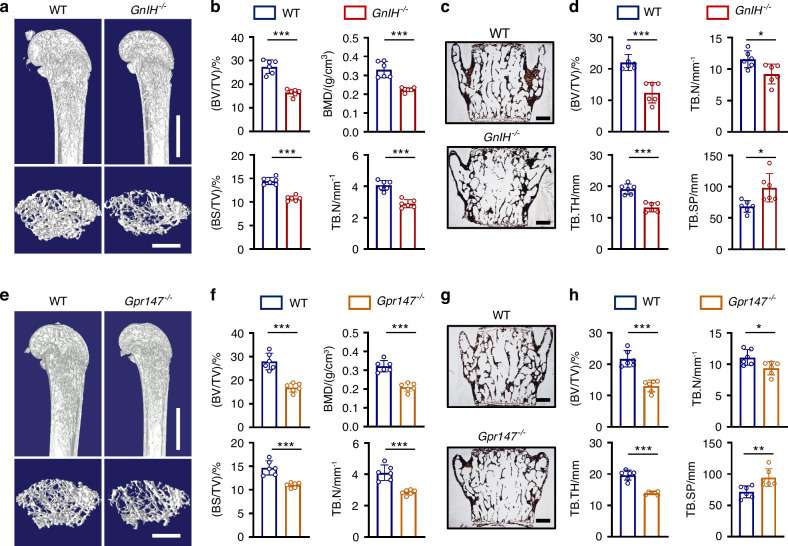
Depletion of GnIH or Gpr147 decreases bone mass and leads to osteoporosis in mice. **a** Representative micro-CT images of femur (top; scale bars: 1 mm) and trabecular bone of the (bottom; scale bars: 500 μm) from 2-month-old male WT and *GnIH*^*−/−*^ male mice. **b** Quantitation of femur trabecular bone parameters from WT and *GnIH*^*−/−*^ male mice. *n* = 6 each group. (***P < 0.001). **c**, **d** The von Kossa staining images of lumbar sections from 2-month-old WT and *GnIH*^*−/−*^ male mice, and trabecular bone parameters were quantified. Scale bars: 500 μm, *n* = 6 each group. (*P < 0.05, ***P < 0.001). **e** Representative micro-CT images of femur (top; scale bars: 1 mm) and trabecular bone (bottom; scale bars: 500 μm) from 2-month-old male WT and *Gpr147*^−/−^ mice. **f** Quantitation of femur trabecular bone parameters from WT and *Gpr147*^−/−^ mice. *n* = 6 each group. (***P < 0.001). **g**, **h** The von Kossa staining images of lumbar sections from 2-month-old WT and *Gpr147*^−/−^ male mice, and trabecular bone parameters were quantified. Scale bars: 500 μm. *n* = 6 each group. (*P < 0.05, **P < 0.01, ***P < 0.001)

To explore whether the novel reproductive hormone GnIH plays a role in regulating bone homeostasis, we created GnIH knockout mice (*GnIH*^*−/−*^ mice). We first confirmed the knockout efficiency of GnIH and observed that the deletion of GnIH does not affect the body weight or size of the mice (Fig. S1A, B). Then, bone mass of femurs in 2-month-old mice was measured using micro-CT. Our results revealed significant decreases of BMD, trabecular bone volume/total volume (BV/TV), external surface of the trabeculae/total sample volume (BS/TV), trabecular number (Tb.N), and trabecular thickness (Tb.Th), but an increase of trabecular separation (Tb.Sp) in *GnIH*^*−/−*^ compared to wild-type (WT) mice (Fig. 1a, b; Fig. S1C–E). Similar results were obtained from Von Kossa staining of lumbar vertebrae (Fig. 1c, d). To investigate whether GnIH exerts its regulatory function in osteoporosis by binding to its receptor Gpr147, we analyzed the bone density and trabecular bone parameters in femur and lumbar vertebrae from Gpr147 knockout mice (*Gpr147*^−/−^ mice). The results were similar to *GnIH*^*−/−*^ mice (Fig. 1e–h; Fig. S1F–J; Fig. S2A–D). Collectively, these data reveal that GnIH/Gpr147 signaling has plays a crucial role in regulating bone mass.

### Deletion of GnIH or Gpr147 accelerates bone loss through enhancing osteoclast number and activity, but has little effect on osteoblast number and activity

**Fig. 2:**
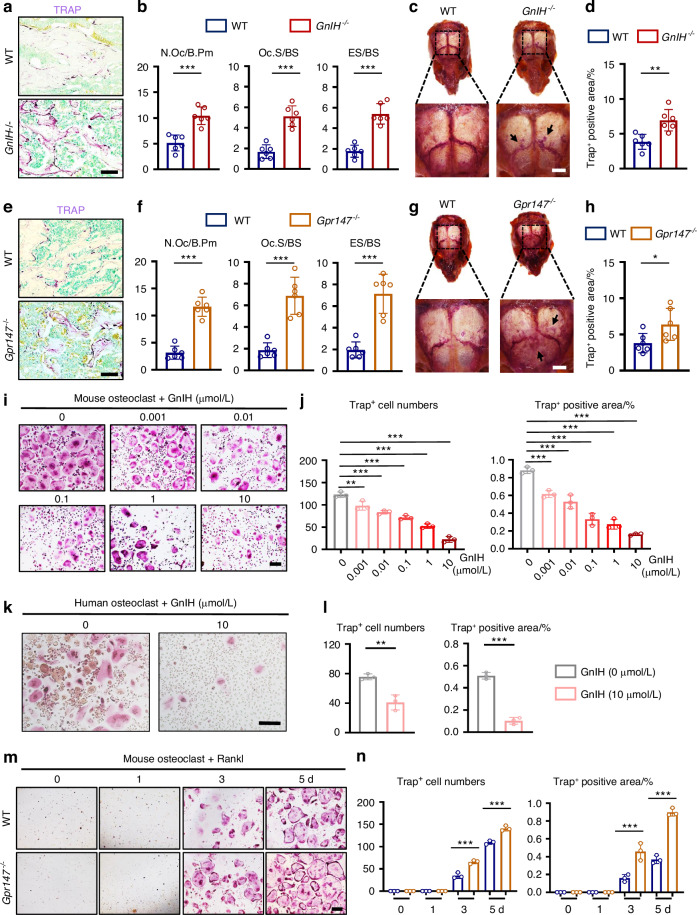
GnIH and Gpr147 negatively regulates osteoclast activity. **a**, **b** Representative TRAP staining images from *GnIH*^*−/−*^ male mice. Scale bars: 100 μm. The number of N.Oc/B.Pm, Oc.S/BS and ES/BS were quantified. *n* = 6 each group. (***P < 0.001). **c**, **d** Representative TRAP staining images in skulls from *GnIH*^*−/−*^ male mice, Scale bars: 2 mm. Trap positive area in each skull was measured and compared. *n* = 6 each group. (**P < 0.01). **e**, **f** Representative TRAP staining images from *Gpr147*^−/−^ male mice. Scale bars: 100 μm. The number of N.Oc/B.Pm, Oc.S/BS and ES/BS were quantified. *n* = 6 each group. (***P < 0.001). **g**, **h** Representative TRAP staining images in skull from *Gpr147*^−/−^ male mice, Scale bars: 2 mm. Trap positive area in skull were measured and compared. *n* = 6 each group. (*P < 0.05). **i**, **j** Representative TRAP staining images of the differentiation of BMMs into osteoclasts in the presence of different doses of GnIH for 6 days. Trap^+^ cell numbers and Trap positive area were measured. *n* = 3 each group. Scale bars: 200 μm. (***P < 0.001). **k**, **l** Representative TRAP staining images of the differentiation of human PBMCs into osteoclasts for 7 days. Trap^+^ cell numbers and Trap positive area were measured. *n* = 3 each group. Scale bars: 200 μm. (**P < 0.01, ***P < 0.001). **m**, **n** Representative TRAP staining images of the differentiation of *Gpr147*^−/−^ BMMs into osteoclasts for indicated times. Trap^+^ cell numbers and Trap positive area were measured. *n* = 3 each group. Scale bars: 200 μm. (***P < 0.001)

To explore the cause of the decline in bone mass of *GnIH*^*−/−*^ or *Gpr147*^*−/−*^mice, we first examined osteoclasts. The activity of osteoclast was significantly enhanced in femurs of *GnIH*^*−/−*^ mice as shown by TRAP staining and TRAP immunofluorescence staining, with the number of osteoclasts (N.Oc)/bone perimeter (N.Oc/N.Oc/B.Pm), osteoclast surface/bone surface (Oc.S/BS) and the eroded surface/bone surface (ES/BS) all increased compared to WT mice (Fig. 2a, b; Fig. S3A, B). Similar results were found in the skulls of *GnIH*^*−/−*^mice (Fig. 2c, d). Deletion of the receptor Gpr147 similarly enhanced osteoclast activity and eroded bone surface in vivo (Fig. 2e–h; Fig. S3C, D). To further examine whether GnIH directly regulated osteoclast differentiation, we evaluated the differentiation of BMMs towards osteoclasts after GnIH treatment. Remarkably, we observed a dose-dependent inhibition of osteoclast differentiation upon GnIH stimulation (Fig. 2i, j). Moreover, our research has shown that GnIH serves as a robust inhibitor of human osteoclast differentiation (Fig. 2k, l). However, GnIH exerted minimal impact on the proliferation and migration of BMMs (Fig. S4A–C). Similarly, knockout of *Gpr147* significantly promotes osteoclast differentiation and maturation (Fig. 2m, n), but has little effect on migration and proliferation of BMMs (Fig. S4D–F). Furthermore, *Gpr147*^*−/−*^osteoclasts express high levels of osteoclastogenic gene transcripts when compared with control osteoclast (Fig. S4G). Moreover, osteoclasts lacking Gpr147 were no longer able to inhibit osteoclast differentiation by GnIH (Fig. S4H, I). Together, all these results confirm that knocking out *GnIH* can enhance osteoclast number, differentiation and activity through Gpr147.

We next explored whether knocking out of GnIH or Gpr147 affected bone formation and osteoblast. Our data showed that the bone formation rate (BFR/BS), the Mineral Apposition Rate (MAR), the number of osteoblasts (N.Ob)/bone perimete (N.Ob/B.Pm), surface area of osteoblasts/bone surface (Ob.S/BS) and the osteoid surface/bone surfac (OS/BS) all have little effect on bone formation and osteoblast in lumbar vertebrae by calcein double labeling assay and Goldner’s staining in *GnIH* and *Gpr147* knockout mice (Fig. S5A–H).

All these results substantiate that the osteoporosis resulting from the knockout of GnIH and Gpr147 is primarily attributed to the activation of osteoclasts and promotion of bone resorption.

### GnIH derived from bone tissue can regulate osteoclastogenesis

**Fig. 3:**
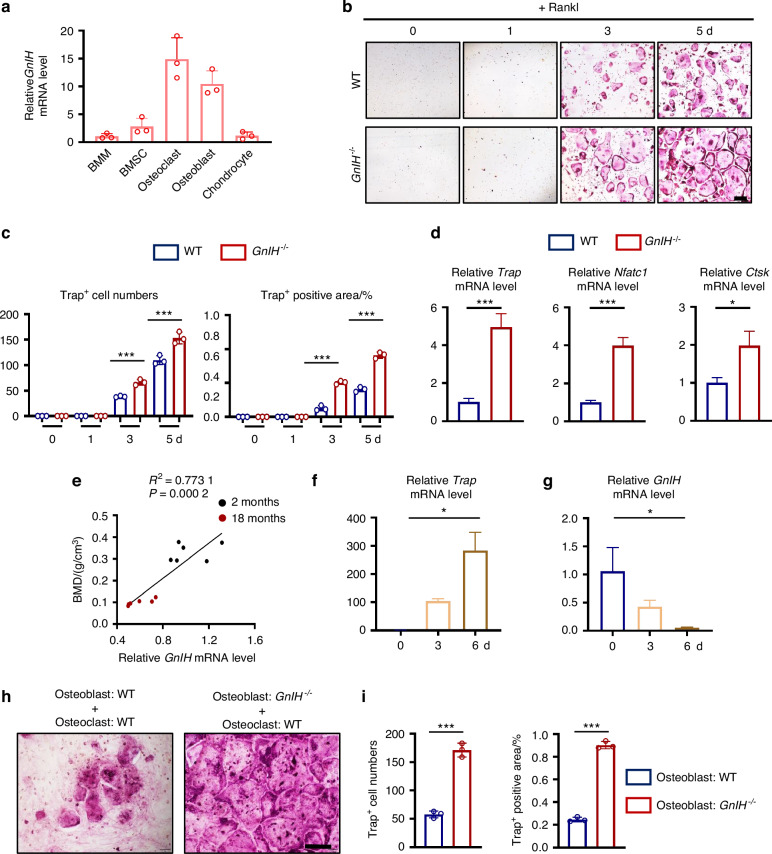
GnIH derived from osteoclasts and osteoblasts negatively regulates osteoclasts activity. **a** RT-qPCR analysis of the expression of *GnIH* in cells of different bone tissues. *n* = 3 each group. **b**, **c** Representative TRAP staining images of the differentiation of *GnIH*^*−/−*^ BMMs into osteoclasts for indicated times. Trap^+^ cell numbers and area were measured. *n* = 3 each group. Scale bars: 200 μm. (***P < 0.001). **d** RT-qPCR analysis of the expression of *Trap*, *Nfatc1* and *Ctsk* in *GnIH*^*−/−*^ and WT osteoclasts. *n* = 3 each group. (*P < 0.05, ***P < 0.001). **e** Correlation between BMD of mice at different ages and the transcriptional expression of GnIH by osteoclasts collected at different ages (2 months or 18 months). The data were analyzed using a Pearson’s correlation coefficient assay (*n* = 12). **f**, **g** RT-qPCR analysis of the expression of *Tra*p and *GnIH* in osteoclasts at indicated times. *n* = 3 each group. (*P < 0.05). **h**, **i** Representative TRAP staining images of the differentiation of BMMs into osteoclasts cultured with WT or *GnIH*^*−/−*^ osteoblasts. *n* = 3 each group. Scale bars: 200 μm. Trap^+^ cell numbers and area were measured. (***P < 0.001)

Our data reveals a high expression of GnIH in bone tissue (Fig. S6A). To investigate which type of bone cells GnIH originates from, we randomly selected BMM as a control group, our RT-qPCR analysis in all bone cells showed that GnIH has strong expression in osteoclast and osteoblast, while it has limited expression in BMM, BMSC and chondrocyte (Fig. 3a). To examine whether the bone-tissue-derived GnIH regulates osteoclast differentiation, we analyzed osteoclastic differentiation using WT and *GnIH*^*−/−*^ BMM. Our results showed that knockout of *GnIH*^*−/−*^ largely enhanced osteoclastic differentiation and formation (Fig. 3b, c), as demonstrated by the differentiation markers *Trap*, *Ctsk*, *Nfatc1* (Fig. 3d). However, here was no difference between WT and *GnIH*^*−/−*^ BMMs in the level of cell proliferation and migration (Fig. S6B–D), suggesting that, besides hypothalamus, osteoclast derived GnIH could regulate osteoclast differentiation.

To further evaluate the function of GnIH in osteoclasts, taking consideration of age and age-induced bone phenotype, we analyzed the GnIH expression of osteoclasts derived from WT mice at different ages (2 months and 18 months) and its correlation with BMD of these mice. Our results showed that expression of *GnIH* in mouse osteoclasts is positively correlated with BMD (*P* = 0.002, *R*^*2*^ = 0.773 1, *n* = 12) (Fig. 3e). We also observed that *GnIH* expression level were inversely correlated with osteoclast differentiation and *TRAP* expression level (Fig. 3f, g). We further examined whether the osteoblast-derived GnIH have regulatory effect on osteoclastogenesis by osteoblast-osteoclast co-culture experiment. Strikingly, the results showed that loss of GnIH in osteoblasts promoted osteoclast differentiation (Fig. 3h, i).

In addition, we found that the relative expression of Rankl at the mRNA level was increased, while the OPG expression was decreased, resulting in an enhanced ratio of Rankl to OPG in *GnIH*^*−/−*^ and *Gpr147*^*−/−*^osteoblasts (Fig. S6E, F). To investigate whether the loss of Gpr147 in osteoblasts has an effect on osteoclast differentiation, we further performed a *Gpr147*^*−/−*^ osteoblast - WT osteoclast co-culture experiment. The results showed that knockout of Gpr147 in osteoblasts promoted the differentiation of BMMs into osteoclasts (Fig. S6G, H). Together, these results indicate that osteoblast or osteoclast secreted GnIH to affect osteoclast formation.

### GnIH/Gpr147 regulates osteoclast differentiation through the PI3K/AKT, MAPK, NF-κB and Nfatc1 signaling pathways

**Fig. 4:**
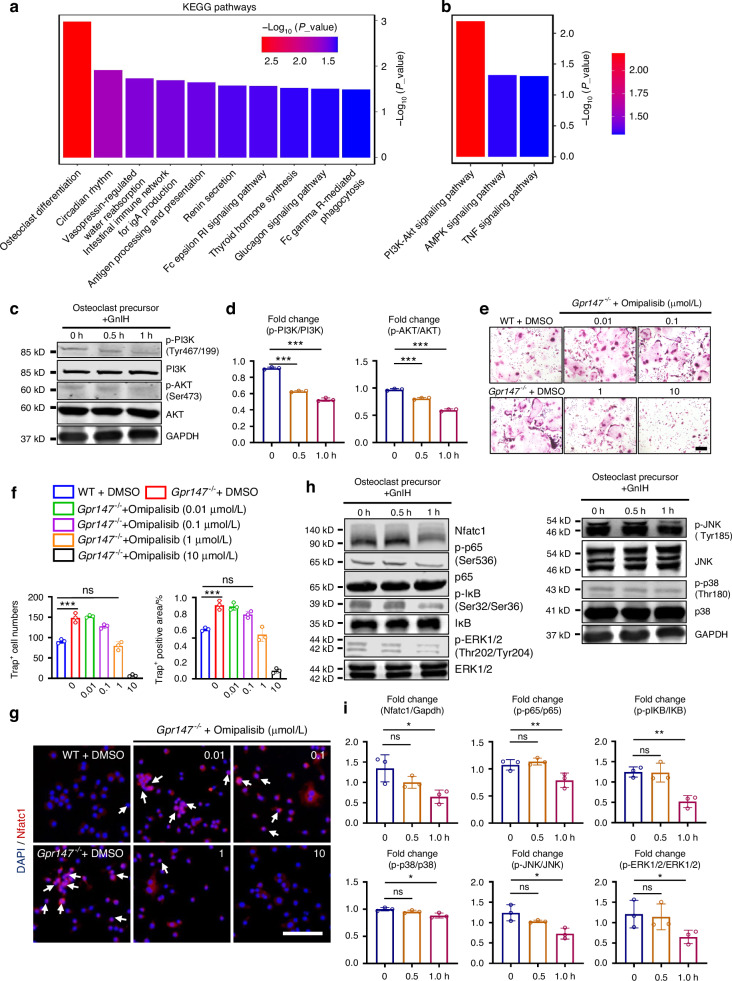
GnIH/Gpr147 regulates osteoclast function through the PI3K/AKT, MAPK, NF-κB and Nfatc1 signaling pathway. **a**, **b** KEGG pathway analysis of the differentiation of WT and *Gpr147*^−/−^ BMMs into osteoclast precursors for 48 h followed by RNA-sequence analysis, *n* = 3 each group. **c**, **d** The protein levels of PI3K/AKT signaling pathway in WT osteoclast precursors treated with 10 μmol/L GnIH for the indicated time periods were measured by western blotting. *n* = 3 each group. (***P < 0.001). **e**, **f** Representative TRAP staining images and quantification of the differentiation of *Gpr147*^−/−^ BMMs into osteoclast cultured with Omipalisib for 5 days. Scale bars: 200 μm. Trap^+^ cell numbers and area were measured. *n* = 3 each group. (***P < 0.001). **g** Representative Nfatc1 immunofluorescence staining images of osteoclast precursors treated with Omipalisib. *n* = 3 each group. Scale bars: 200 μm. **h**, **i** The protein levels of NF-κB, MAPK and Nfatc1 signaling pathway in WT osteoclast precursors treated with 10 μmol/L GnIH for the indicated time periods were measured by western blotting. *n* = 3 each group. (*P < 0.05, **P < 0.01, ***P < 0.001)

To investigate how GnIH/Gpr147 affects osteoclastogenesis, we performed RNA sequencing analysis on osteoclast precursors (BMM cells after two-day differentiation) from Gpr147-deficient and WT mice. Not surprisingly, the pathway associated with osteoclast differentiation was enriched and ranked first in Gpr147-deficient osteoclast precursors (Fig. 4a). Moreover, KEGG analysis revealed that deletion of Gpr147 led to significant alterations in the phosphatidylinositol 3-kinase (PI3K)-AKT pathway in osteoclast precursor cell (Fig. 4b). To validate that GnIH induced osteoclast differentiation via PI3K-AKT signaling, we examined the phosphorylation levels of PI3K and AKT at various time points (0, 0.5, and 1 h) in GnIH-stimulated osteoclast precursors. We found that phosphorylation of PI3K and AKT were inhibited in a time-dependent manner after GnIH stimulation (Fig. 4c, d). Furthermore, we employed the inhibitors of PI3K/AKT signaling during osteoclast differentiation. Our data showed that *Gpr147* knockout dramatically induced osteoclast differentiation, while the two inhibitors of the PI3K/AKT signaling pathway (Omipalisib and 3-MA) significantly suppressed *Gpr147* knockout-induced osteoclast differentiation (Fig. 4e, f; Fig. S7A, B). Because Nfatc1 nuclear translocation is the downstream events of PI3K/AKT signaling during osteoclast differentiation,^39–42^ we next examined whether GnIH or the PI3K/AKT signaling inhibitors have regulatory effects on Nfatc1 nuclear translocation. The results showed that GnIH significantly suppressed the nuclear translocation of Nfatc1 (Fig. S7C, D), whilst the two PI3K/AKT signaling inhibitors can reduce Nfatc1 nuclear translocation after knockout of Gpr147 (Fig. 4g; Fig. S7E–G). Together, our result indicated that GnIH/Gpr147 regulates osteoclast function through the PI3K/AKT signaling.

There is abundant evidence supporting that MAPK, NF-κB and Nfatc1 signaling pathways are crucial in regulating osteoclast differentiation. To investigate whether Gpr147 also regulates osteoclast differentiation via MAPK, NF-κB and Nfatc1 signaling pathways, we conducted additional analyses. A time-dependent inhibition of phosphorylation on critical proteins such as p65, IκB, p38, JNK, and ERK follows GnIH stimulation, coinciding with a decreased expression of Nfatc1 (Fig. 4h, i). Thus, our data provides compelling evidence showing that GnIH/Gpr147 regulates osteoclast function through the PI3K/AKT, MAPK, NF-κB and Nfatc1 signaling pathways.

### GnIH inhibits bone loss induced by aging, OVX and LPS

**Fig. 5:**
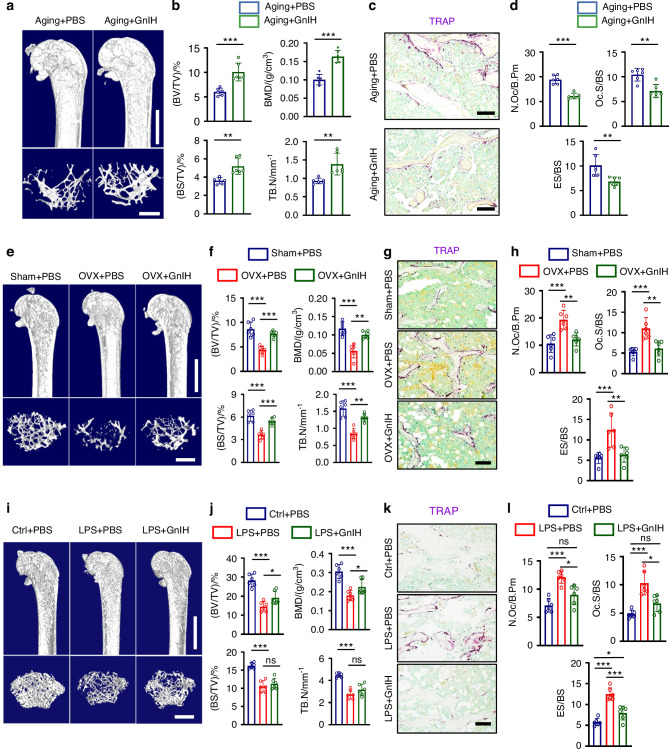
GnIH prevents aging-, OVX-, and LPS-induced bone loss. **a**, **b** Representative micro-CT images of femurs (top; scale bars:1 mm) and trabecular bone (bottom; scale bars: 500 μm) from PBS or 0.1 mg/kg GnIH treatment 18-month-old male mice for 30 days. Quantitation of femur trabecular bone parameters from PBS or 0.1 mg/kg GnIH treatment 18-month-old male mice for 30 days. *n* = 6 each group. (**P < 0.01, ***P < 0.001). **c**, **d** Representative TRAP staining images from 0.1 mg/kg GnIH treatment 18-month-old male mice.Scale bars, 100 μm. The number of N.Oc/B.Pm, Oc.S/BS and ES/BS were quantified. *n* = 6 each group. (**P < 0.01, ***P < 0.001). **e**, **f** Representative micro-CT images of femurs (top; scale bars: 1 mm) and trabecular bone (bottom; scale bars: 500 μm) from Sham, OVX or 0.1 mg/kg GnIH treatment OVX mice for 30 days. Quantitation of femur trabecular bone parameters from Sham, OVX or 0.1 mg/kg GnIH treatment OVX mice for 30 days. *n* = 6 each group. (**P < 0.01, ***P < 0.001). **g**, **h** Representative TRAP staining images from 0.1 mg/kg GnIH treatment OVX mice. Scale bars, 100 μm. The number of N.Oc/B.Pm, Oc.S/BS and ES/BS were quantified. *n* = 6 each group. (**P < 0.01, ***P < 0.001). **i**, **j** Representative micro-CT images of femurs (top; scale bars:1 mm) and trabecular bone (bottom; scale bars: 500 μm) from 0.1 mg/kg GnIH treatment LPS-induced male mice for 7 days. Quantitation of femur trabecular bone parameters from 0.1 mg/kg GnIH treatment LPS-induced male mice for 7 days. *n* = 6 each group. (*P < 0.05, ***P < 0.001). **k**, **l** Representative TRAP staining images from 0.1 mg/kg GnIH treatment LPS-induced male mice. Scale bars, 100 μm. The number of N.Oc/B.Pm, Oc.S/BS and ES/BS were quantified. *n* = 6 each group. (*P < 0.05, ***P < 0.001)

To further validate whether GnIH has therapeutic effect on osteoporosis, we utilized three distinct mouse models to mimic aging-, postmenopause-, inflammation- induced osteoporosis to validate this function. First, we treated aging mouse with GnIH (0.1 mg/kg/day). GnIH treatment resulted in increased bone mass and BMD in 18-month-old mice (Fig. 5a, b, Fig. S8A), as well as in significantly inhibited osteoclast number and activity (Fig. 5c, d). Importantly, GnIH treatment had no evidence of toxicity or adverse effects in heart, liver, spleen, lung, and kidney tissues compared to the control group (Fig. S8B). It has been shown that IL-1β is upregulated in the production of chronic inflammatory factors during aging.^43^ Therefore, we evaluated whether GnIH affects IL-1β-induced osteoclast differentiation and formation in vitro. GnIH treatment suppressed osteoclast activity in an IL-1β inflammatory environment (Fig. S8C, D).

Next, we evaluated GnIH effect in OVX osteoporosis mouse model. After one month of daily intraperitoneal injection of GnIH, osteoclast activity was remarkably inhibited and bone loss was rescued in OVX mice (Fig. 5e–h; Fig. S8E, F). Surprisingly, the value of BMD and BV/TV in GnIH treatment group were almost close to the Sham operation group, suggesting that the GnIH could exert strong therapeutic effect on osteoporosis.

Last, using the LPS induced inflammatory bone loss mouse model, we evaluated the effect of GnIH on LPS-induced bone loss. Consistently, bone mass significantly increased while osteoclasts were inhibited after GnIH treatment (Fig. 5i–l; Fig. S9A–C). Furthermore, GnIH treatment suppressed LPS-induced osteoclast differentiation *ex vitro* (Fig. S9D, E), and the serum inflammatory factor TNF-α and IL-1β were mildly decreased, whereas the level of IL-6 remains unchanged (Fig. S9F). These results suggested that in LPS-induced mice, GnIH treatment both affects inflammatory factor levels and slightly improves the inflammatory milieu, while also strongly inhibiting osteoclast activity. This dual action contributes to the preservation of bone mass and halting bone loss.

Taken together, these findings provide compelling evidence that GnIH treatment effectively enhances bone mass and inhibited osteoclast activity in aging, OVX, and LPS-induced bone loss models.

### Green light promotes GnIH release and alleviates bone loss in OVX-induced osteoporosis mice

**Fig. 6:**
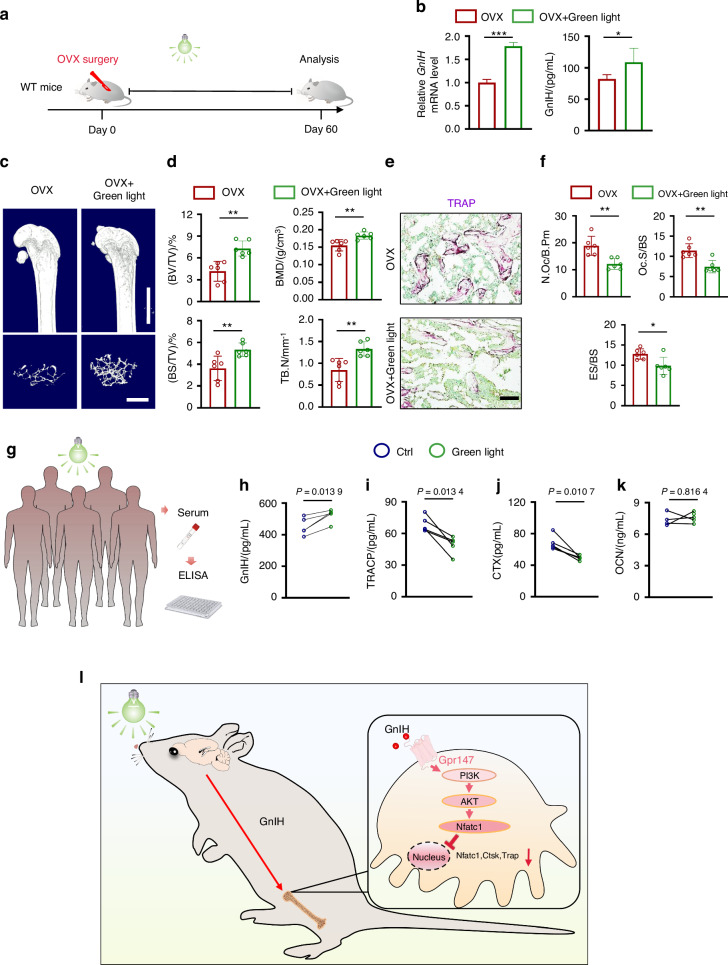
Green light therapy promotes GnIH release and rescues bone loss in OVX mice. **a** OVX mice were exposed to green (520–525 nm, 400 Lux) light therapy for 8 h (8:00 am-16:00 pm) for 60 days. **b** RT-qPCR analysis of the expression of GnIH in the hypothalamus of OVX mice with or without exposure to green light (left). *n* = 3 each group. Serum GnIH content was detected by ELISA (right). *n* = 6 each group. (*P < 0.05, ***P < 0.001). **c**, **d** Representative micro-CT images of femurs (top; scale bars:1 mm) and trabecular bone (bottom; scale bars: 500 μm) from OVX mice with or without green light exposure. *n* = 6 each group. (**P < 0.01). **e**, **f** Representative TRAP staining images from OVX mice with or without green light exposure. Scale bars, 100 μm.The number of N.Oc/B.Pm, Oc.S/BS and ES/BS were quantified. *n* = 6 each group. (*P < 0.05, **P < 0.01). **g** Five subjects were exposed to green (520–525 nm, 400–1000 Lux) light therapy for 2 h (9:00 am-11:00 am) for 7 days. **h**–**k** Human serum GnIH, bone formation markers (OCN), bone resorption markers (TRACP and CTX) content was detected by ELISA. *n* = 5. Paired t-test. **l** Green light therapy promotes the synthesis and secretion of GnIH in hypothalamus. GnIH is then transported to bone tissue through circulation, binds to osteoclast Gpr147, and inhibits osteoclast differentiation

Green light can promote GnIH expression and synthesis in hypothalamus in poultry animals,^36,37^ and green light therapy has demonstrated analgesic effects in both human and mice.^38^ Therefore, we hypothesized that green light exposure can promote GnIH release and prevent bone loss. To verify the therapeutic effect of green light, we exposed OVX mice to green light for 8 h (8:00 am-16:00 pm) per day for 60 days (Fig. 6a). We evaluated the hypothalamic GnIH transcription level in mice, and the results showed that exposure to green light promoted the transcriptional expression of hypothalamic *GnIH* (Fig. 6b). Consequently, serum GnIH levels were also increased after green light exposure (Fig. 6b). Furthermore, bone mass were significant increased and osteoclast number and activity were decreased in OVX mice when exposed to green light compared to OVX control group (Fig. 6c–f; Fig. S10A). These results indicate that exposure to green light stimulated the release of GnIH and effectively mitigated bone loss in OVX mice. We conducted further evaluation of the changes in bone mass following green light therapy in *GnIH*^*−/−*^ and *Gpr147*^*−/−*^ mice, and the data showed that green light treatment can only partially improve the bone parameters of *GnIH*^*−/−*^ and *Gpr147*^*−/−*^ mice (Fig. S11A–E), which may be related to the regulation of melatonin-GnIH pathway by green light.^36,37^

To examine the prospective utility of green light therapy as a novel clinical approach for treating osteoporosis, we conducted a study involving five healthy adult participants. Each participant underwent a one week of daily green light phototherapy sessions, lasting 2 h per day (Fig. 6g). We evaluated the serum GnIH levels, bone resorption markers and bone formation marker of subjects before and after green light therapy. The results revealed a significant elevation in serum GnIH levels following green light therapy (Fig. 6h). While the serum levels of TRACP and CTX (tartrate acid phosphatase, type I collagen carboxy-terminal peptide, bone resorption markers) were observed to be down-regulated (Fig. 6i, j), and the level of OCN (osteocalcin, bone formation markers) did not change significantly after green light therapy (Fig. 6k). Furthermore, healthy adult participants exposed to regular light showed no significant changes in serum GnIH, OCN, TRACP and CTX (Fig. S12A–D). Our human study was consistent with our animal study and demonstrates the potential of green light therapy in suppressing osteoporosis.

## Discussion

The regulatory mechanism that interlinks reproductive hormones and bone homeostasis continues to be an area of ambiguity. For the first time, our study presents groundbreaking evidence demonstrating the involvement of GnIH, a crucial upstream reproductive hormone, in the regulation of bone mass. Our finding revealed that GnIH significantly enhance the function of osteoclasts in vivo and in vitro by inhibiting the receptor Gpr147. In addition, we discovered that both osteoblasts and osteoclasts possess the capability to secrete GnIH, and demonstrated its importance in GnIH/Gpr147 regulation of bone homeostasis. Mechanistically, we demonstrated that GnIH/Gpr147 affects osteoclast differentiation by regulating the PI3K/AKT, MAPK, NF-κB and Nfatc1 signaling pathways. Finally, we found that green light exposure can promote GnIH release and can avoid OVX mice bone loss, and green light therapy significantly downregulated serum levels of bone resorption markers in humans, providing a novel approach for the treatment of osteoporosis disease (Fig. 6l).

Our study further demonstrates that the mechanism of green light therapy in rescuing bone loss in OVX mice probably lies in reducing osteoclast activity through regulating GnIH release, which originates from the hypothalamus and bone tissue. Traditionally, ultraviolet light and infrared light plays a crucial role in bone mass regulation.^44–46^ However, it is important to note that prolonged ultraviolet therapy can cause skin aging and increase the risk of genetic mutations.^47^ Similarly, while far-infrared light may have potential benefits, excessive exposure can damage the skin and eyes.^48^ In comparison, green light is generally considered safer and less harmful to the skin and eyes.^49^ In addition, while green light laser therapy has been shown to promote the differentiation and proliferation of mesenchymal stem cells into osteoblasts, relevant studies have only been conducted at the cellular level, which does not necessarily imply efficacy in vivo. Significantly, our study provides evidence that green light therapy can effectively modulate the levels of bone resorption markers in human serum. This discovery introduces a novel and practical approach that holds promise for the clinical treatment of osteoporosis. However, more detailed investigations into how green light enhances the release of GnIH are needed in the future.

GnRH agonist therapy is commonly utilized in the treatment of certain cancers and individuals with precocious puberty.^8,50^ However, data suggest a potential risk of reduced bone mass following GnRH therapy. As reported, GnIH plays a regulatory role in the synthesis and secretion of GnRH, acting as an inhibitor of GnRH secretion.^13^ It has been reported that GnIH neurons regulate not only GnRH but also Kisspetin, which is also believed to be a key reproductive hormone upstream of GnRH.^9,32^ Our study has revealed a significant regulatory effect of GnIH on bone mass. Furthermore, our findings may provide an explanation for the association between excessive activation of GnRH and low bone mass. Inadequate synthesis of GnIH may potentially contribute to the increased risk of low bone mass associated with GnRH agonist therapy.

The level of GnIH expression has been linked to abnormal adolescent development and reproductive dysfunction.^9^ The loss of GnIH expression is believed to be a contributing factor to the onset of precocious puberty.^34^ It is also believed that GnIH antagonists could be developed into drugs for reproductive dysfunction, and whether fertility drugs developed for GnIH/Gpr147 increase the risk of osteoporosis needs to be studied and noted.^33,51^ In our study, we conducted long-term green light therapy on OVX mice, exposing them to up to 8 h per day. It is worth noting that mice are nocturnal animals,^52^ and such prolonged exposure to green light therapy may potentially impact their biological rhythms.^53^ Therefore, the appropriate duration and intensity of green light illumination in clinical translational therapy require further investigation and discussion. Additionally, the response time to green light therapy may vary among different populations and should be explored further.^54^

In total, our data strongly indicates the role of the key upstream reproductive hormone, GnIH, in regulating bone homeostasis. This study not only advances our understanding of this mechanism but also provides a novel idea and potential method for clinical treatment and prevention of osteoporosis.

## Materials and methods

### Mouse model

All experimental processes comply with East China Normal University (ECNU) animal ethics (ethical approval number m20230206). ECNU Animal Center provides 8–12 week old or 18 months old WT C57BL/6J mice. It was established that all mice were maintained over a period of 12 h under a complete dark-light cycle (with a white illumination level of 200 lux) as well as a constant temperature and humidity level (20–26 °C), unless otherwise indicated.

*GnIH*^−/−^ mice and *Gpr147*^−/−^ mice were generated by Cyagen using the CRISPR/Cas9 system in the C57BL/6N mouse strain. To create a GnIH knockout mouse, Exons 1 to 3 were targeted, encompassing a 567 bp coding sequence within the region. Specifically, gRNA1 (GATATTCTATACACGCTAGCTGG) targets exon 1, and gRNA2 (CTTCTCCAGACCTAGTGAACAGG) targets exon 3. For the creation of a Gpr147 knockout mouse, Exons 2 to 3 were targeted, covering a 415 bp coding sequence. Notably, gRNA3 (AGCCCAAGCACTTTCGAAGGTGG) targets exon 2, while gRNA4 (CATGCAGACGGAGTAAAGCCAGG) targets exon 3.

Genotype identification of mice was performed using the following primer sequences: *GnIH*^−/−^-F: CATTTGCCAAATTAGACCCTTAGGG, *GnIH*^−/−^-R: AAATGCAACCCAGGGTTGATGTC, *GnIH*^−/−^-He/Wt-F: AGCCCGACTTCAAGAGGCTAC; *Gpr147*^−/−^-F: GTGGACAGTAATAAGTGGGCTTAGGGT, *Gpr147*^−/−^-R: AGCTAAACAACAGTCTCCTGCATG, *Gpr147*^−/−^-He/Wt-F: GTAATTCTGGGACTGGCACGC.

### Cell culture and osteoclast differentiation

Refer to our previous research reports,^6,55,56^ bone marrow macrophages (BMMs) were obtained from the femurs and tibias of 8-week-old WT, *GnIH*^−/−^ or *Gpr147*^−/−^ mouse. They were then cultured in α-MEM medium (Gibco) with the addition of 10 ng/mL M-CSF (R&D), 10% FBS (Gibco), and 1% penicillin-streptomycin (HyClone). For osteoclast differentiation assay, BMMs were plated in 96-well plates at a density of 1 × 10^4^ cells per well. Subsequently, they were stimulated with 50 ng/mL of RANKL (R&D) and 10 ng/mL of M-CSF (R&D). After 5–7 days, The TRAP staining kit (Sigma-Aldrich) was used to identify TRAP^+^ osteoclasts (five or more nuclei) from osteoclasts fixed in 4% Paraformaldehyde (PFA). For LPS-induced osteoclast differentiation assay, BMMs were plated in 96-well plates at a density of 1 × 10^4^ cells per well. The cells were incubated with 50 ng/mL of RANKL (R&D), 10 ng/mL of M-CSF (R&D), 50 ng/mL of LPS (Beyotime) and 10 μmol/L GnIH for 5 days. After fixed in 4% PFA, the cells were stained for TRAP assay.^57–59^

### Osteoblast-Osteoclast co-culture

Isolated preosteoblastic cells from the calvaria of 3–5-day-old neonatal mice. Co-culturing was carried out in 96-well plates with BMMs (1 × 10^4^ cells per well) and preosteoblastic cells (1.5 ×10^3^ cells per well) in α-MEM (Gibco) (10% FBS), 100 ng/mL 1 alpha, 25-dihydropyrene (HyClone), 1% penicillin-streptomycin. One week later, TRAP staining was performed, and the number of osteoclasts exhibiting 5 or more nuclei and positive for TRAP staining was quantified.

### CCK-8 assay

After seeding 96-wells with a combination of 100 μL α-MEM and 20 ng/mL M-CSF-containing BMMs (1.2 ×10^4^ cells per well) for two days, then supplemented with CCK-8 solution (10 μL per well) for 90 min and read the absorbance at 450 nm.

### Cell migration assay

For BMM migration, GnIH at dosages of 0, 0.01, 0.1, 1, 10 μmol/L was added in the lower chamber while BMMs were seeded in the upper chamber (1 × 10^5^ cells per well) of transwell inserts. After one day, the migrated BMMs were fixed in 4% PFA for 0.5 h, then used water wash 4 times and stained them for 2 h with 0.1% crystal violet. In total, four fields of view were photographed per insert and then quantified using Media Cybernetics’ Image-Pro Plus 6.0 (Media Cybernetics).

### Treatment with GnIH peptide in vivo

OVX-induced and LPS-induced (LPS was injected intraperitoneally at a dose of 5 mg/kg body weight on day 0 and day 4) bone loss mouse models were used as previously described.^5,6,60^ Mouse GnIH peptide with the sequence Phe-Pro-Ser-Leu-Pro-Gln-Arg-Phe-NH2 was intraperitoneally injected daily,^61^ at the dosage of 0.1 mg/kg in 200 μL PBS.^62,63^ OVX mice and aging mice were treated with GnIH for one month, LPS-induced mice were treated with GnIH for seven days. GnIH peptide was synthesized by Shanghai Bootech BioScience & Technology.

### Green light exposure

The green LED light source (Shanghai wence) was fixed on the top of a special customized cabinet. OVX mice were subjected to green LED light exposure within a custom-designed cabinet, while being provided unrestricted access to food and water. Green light (520–525 nm, 400 Lux) therapy exposure for 8 h (8:00 am-16:00 pm) daily for 60 consecutive days^38,64^ was applied to treated mice. Following daily green light therapy, the mice were subsequently returned to their standard animal housing facility.

A total of five healthy adult males between the ages of 20 and 40, who do not have any visual impairment or eye diseases, have not taken any hormonal or osteoporosis medications within the past three months, and do not have a history of chronic diseases or other medical conditions, were selected for this study. For human green light or regular light exposure experiment, the subjects were initially exposed to regular light for 7 days as control, followed by green light for another 7 days after a two-week rest period. LED green light source emitting wavelengths between 520–525 nm or regular light (6000 K LED light) were utilized. The experiment took place in their designated room from 9:00 am to 11:00 am daily. The LED green light or regular light source was positioned at a distance of 1-2 m from the subjects’ eyes, and the light intensity ranged from 400 to 1 000 lux. To ensure individual comfort, subjects had the flexibility to adjust the distance between the light source and their eyes within the specified range. During the experiment sessions, subjects were specifically instructed to remain awake and maintain a normal blink rate without directly staring at the light source. When exposed to green light or regular light, each subject wore a hospital gown made of the same material. They were encouraged to engage in activities that did not require additional sources of light including conversation and listening to music, while watching television or using screen devices, were forbidden. Subjects were collected for peripheral venous blood sampling before the experment and after one week of green light or regular light therapy. The experiment underwent thorough review and was approved by the Medical Ethics Committee of Yangzhi Rehabilitation Hospital (Approval No. YZ 2023-070).

### Human peripheral blood mononuclear cells differentiation

Obtaining blood from healthy adult individuals. After ficoll centrifugation, human PBMCs were selected and were plated in 96-well plates at a density of 1.5 ×10^4^ cells per well. Subsequently, they were stimulated with 60 ng/mL of RANKL (R&D) and 20 ng/mL of M-CSF (R&D), while treated with the 10 μmol/L human GnIH with the sequence Val-Pro-Asn-Leu-Pro-Gln-Arg-Phe-NH2. After 7 days, The TRAP staining kit (Sigma-Aldrich) was used to identify TRAP^+^ osteoclasts (five or more nuclei) from osteoclasts fixed in 4% Paraformaldehyde (PFA). Human GnIH peptide was synthesized by Shanghai Bootech BioScience & Technology.

### Western blotting analysis

Following the treatment of cells with GnIH (10 μmol/L), proceed to lyse the cells using RIPA buffer in order to extract proteins. The protein concentration can then be quantified using the BCA assay (Thermo). Electrophoresis and transfer to nitrocellulose filter membranes (Beyotime) were followed by 3 h of treatment with 5% bovine serum albumin (Beyotime), and incubated with specific antibodies: GAPDH antibody (CST), p-PI3K (Tyr467/199) antibody (Abmart), p-AKT (Ser473) antibody (Abmart), p-p65 (Ser536) antibody (Abmart), p-IκB (Ser32/Ser36) antibody (Abmart), p-p38 (Thr180) antibody (Abmart), p-JNK (Tyr185) antibody (Abmart), p-ERK (Thr202/Tyr204) antibody (Abmart), p65 antibody (Abmart), IκB antibody (Abmart), p38 antibody (Abmart), JNK antibody (Abmart), ERK antibody (Abmart), AKT antibody (CST), PI3K antibody (CST), Nfatc1 antibody (SANTA CRUZ). A secondary antibody (Licor) was added to the membranes after overnight incubation at 4 °C. Images were captured using the Odyssey Infrared Imaging System. Image-Pro Plus 6.0 software was used to quantify the bands. First, all readings were normalized to the corresponding band of GAPDH. Next, to calculate the fold changes, p-AKT was normalized to AKT samples, p-PI3K was normalized to PI3K samples, p-p65 was normalized to p65 samples, p-IκB was normalized to IκB samples, p-p38 was normalized to p38 samples, p-JNK was normalized to JNK samples and p-ERK was normalized to ERK samples.

### Immunofluorescence staining

For paraffin sections, after treatment with gradient of dehydration and 20 mg/mL proteinase K for 20 min, paraffin sections were fixed in 2% BSA and 0.1% Triton X-100 buffers for 1 h, they were incubated overnight at 4 °C with first antibody Trap (Novus), then with secondary antibody for 1 h, followed by DAPI staining for nuclei (Sigma).^65^

### RNA and RT-qPCR

RNA and cDNA were extracted separately using Trizol (Invitrogen, USA) and 2× Hifair® II. SuperMix (Yeasen, China). Subsequently, the quantitative real-time PCR (RT-qPCR) reaction was performed using the Hieff® qPCR SYBR® Green Master Mix (YEASEN). Hypothalamic tissue was isolated from the brains of mice treated with green light, and the bone tissue was derived from the femur of the mouse after stripping the muscle tissue. The tissues were snap-frozen using liquid nitrogen and subsequently pulverized. BMMs and BMSC were isolated from 8-week-old WT mice. Osteoblasts and chondrocytes were differentiated from BMSCs, while osteoclasts were differentiated from BMMs.

The PCR primer sequences are as follows: Nfatc1-F: CCCGTCACATTCTGGTCCAT, Nfatc1-R: CAAGTAACCGTGTAGCTGCACAA; Ctsk-F: ATGTGGGTGTTCAAGTTTCTGC, Ctsk-R: CCACAAGATTCTGGGGACTC; Trap-F: CAGCTCCCTAGAAGA TGGATTCAT, Trap-R: GTCAGGAGTGGGAGCCATATG; Actin-F: GTACGCCAACACAGTGCTG, Actin-R: CGTCATACTCCTGCTTGCTG; GnIH-F: CAAGACACCCGCTGATTTGC, GnIH-R: TTCGCTTTCCACCAGGACTC; Gpr147-F: CCGAGTCTGAACGAGAGTGA, Gpr147-R: CGGTTCTTAAGCACGATGAA;

### RNA-sequencing analysis

BMMs were isolated from 8-week-old WT and *Gpr147*^−/−^ mouse and then stimulated with RANKL (50 ng/mL) and M-CSF (10 ng/mL) for 48 h. Cells were lysed using Trozil and sent to Shanghai Origin-gene Biological Company for RNA extraction, sequencing and analysis. Genes exhibiting a false discovery rate (FDR) < 0.05 were classified as differentially expressed.

### Micro-CT

Femurs of mice were fixed in 4% PFA for 48 h and washed with water 4 times, then transferred into 75% alcohol for preservation. Skyscan-1272 micro-CT (Bruker micro-CT, Belgium) was used to examine the bone micro-architecture related parameters including bone mineral density (BMD), bone volume density (BV/TV), bone area density (BS/TV), trabecular number (Tb.N) and trabecular thickness (Tb.Th). The following software was used: Skyscan NRecon software (Bruker), CT Analyser software (Bruker), CT Voxsoftware (Bruker), and scanning parameters and analysis methods were as previously described.^6,7^

### TRAP staining

After treatment with a dehydration gradient, the paraffin sections were treated with 0.1% Triton X-100 for 30 min and washed with PBS 2 times, and then stained with TRAP staining kit (Sigma-Aldrich) at 37 °C for 1 h. The OsteoMeasure Analysis System (Osteometrics) was used to analyze the number, surface area and eroded surface area of osteoclast. For calvarias TRAP staining, calvarias were isolated and fixed in 4% PFA for 24 h, then treated wtih 0.1% Triton X-100 for 1 h and washed with PBS 5 times. After stained with TRAP staining kit at 37 °C for 4 h, the positive area were determined using the Image-Pro Plus 6.0 software.

### Calcein labeling

WT, *GnIH*^−/−^ and *Gpr147*^−/−^ mice were injected with calcein (30 mg/kg) on postnatal day 55 and postnatal day 65, and euthanized on postnatal day 72. Vertebrae were fixed in 4% PFA for 24 h, processed through an alcohol dehydration gradient, then embedded with methyl methacrylate. 5 µm sections were cut for calcein double labeling analysis and Goldner’s staining. For Goldner’s staining, the sections were stained with hematoxylin, Ponceau Acid, Orange G and light green solution. The OsteoMeasure Analysis System (Osteometrics) were used to measure bone formation rate per bone surface, mineral apposition rate, osteoblast number, osteoblast surface area, and osteoids per bone surface.

### Elisa assay

Analysis of GnIH levels in mouse serum by using the KL-GnIH-Mu kit (Kanglang). Analysis of TNF-α, IL-1β, IL-6 levels in mouse serum by using the ELISA Kit (Jingmei). Analysis of GnIH, TRACP, CTX, OCN levels in human serum by using the ELISA Kit (Jingmei).

### Statistical analysis

All data are reported as means ± SD. GraphPad Prism 8.0 was employed to evaluate significant differences in the data. One-way ANOVA followed by Tukey’s *t* tests or two-way ANOVA followed by Tukey’s *t* tests was used for multiple comparisons. When comparing only two groups, paired or unpaired Student’s *t* test was used as appropriate. Statistical significance was *P* < 0.05.

## Supplementary information

GnIH Osteoclast Supplemental Figure Legend

Supplemental Figure 1-12
