# Supplementary material for: GnIH secreted by green light exposure, regulates bone mass through the activation of Gpr147
Source: Bone Res. 2025 Jan 21;13:13. doi: 10.1038/s41413-024-00389-7 (PMC11751147; doi:10.1038/s41413-024-00389-7)
Supplement: Supplementary file 1 — GnIH Osteoclast Supplemental Figure Legend [file 41413_2024_389_MOESM1_ESM.pdf]

Supplemental Figure Legend

Supplemental Figure 1. Knocking out GnIH or Gpr147 significantly decreases trabecular bone mass in male and female mice.

(A) RT-qPCR analysis of the expression of *GnIH* mRNA in *GnIH*$^{-/-}$ and WT BMMs. n = 3 each group. (***P < 0.001).

(B) Quantitation of weight and body length from 2-month-old *GnIH*$^{-/-}$ male mice and WT male mice. n = 6 each group.

(C) Quantitation of femur trabecular bone parameters from male *GnIH*$^{-/-}$ mice. n = 6 each group. (***P < 0.001).

(D) Representative micro-CT images of femurs (top; scale bars: 1 mm) and trabecular bone (bottom; scale bars: 500 μm) from 2-month-old *GnIH*$^{-/-}$ female mice.

(E) Quantitation of femur trabecular bone parameters from *GnIH*$^{-/-}$ female mice. n = 6 each group. (*P < 0.05, **P < 0.01, ***P < 0.001).

(F) RT-qPCR analysis of the expression of *Gpr147* mRNA in *Gpr147*$^{-/-}$ and WT BMMs. n = 3 each group. (*P < 0.05).

(G) Quantitation of weight and body length from 2-month-old *Gpr147*$^{-/-}$ male mice and WT male mice. n = 6 each group.

(H) Quantitation of femur trabecular bone parameters from *Gpr147*$^{-/-}$ male mice. n = 6 each group. (*P < 0.05, ***P < 0.001).

(I) Representative micro-CT images of femurs (top; scale bars:1 mm) and trabecular

bone (bottom; scale bars: 500 μm) from 2-month-old *Gpr147*$^{-/-}$ female mice.

(J) Quantitation of femur trabecular bone parameters from *Gpr147*$^{-/-}$ female mice. n = 6 each group. (**P < 0.01, ***P < 0.001).

Supplemental Figure 2. **Knocking out *GnIH* or *Gpr147* showed reduced trabecular area.**

(A and B) Representative HE staining of femurs from 2-month-old *GnIH*$^{-/-}$ male mice and trabecular bone area were counted. n = 5 each group. Scale bars: 100 μm. (***P < 0.001).

(C and D) Representative HE staining of femurs from 2-month-old *Gpr147*$^{-/-}$ male mice and calculate trabecular bone area were counted. n = 5 each group. Scale bars: 100 μm. (***P < 0.001).

Supplemental Figure 3. Knocking out *GnIH* or *Gpr147* affects bone resorption but not formation *in vivo*.

(A and B) TRAP immunofluorescence staining images from *GnIH*$^{-/-}$ male mice or WT male mice, and TRAP^{+} osteoclast area in trabecular bone were measured. Scale bars: 100 μm. n = 6 each group. (***P < 0.001).

(C and D) TRAP immunofluorescence staining images from *Gpr147*$^{-/-}$ male mice or WT male mice, and TRAP^{+} osteoclast area in trabecular bone were measured. n= 3

each group. Scale bars: 100 μm. n = 6 each group. (***P < 0.001).

Supplemental Figure 4. GnIH inhibits BMM differentiation into osteoclasts by activating Gpr147, but does not affect cell proliferation and migration.

(A and B) Representative images of BMMs from WT mice subjected to the Transwell migration assay. BMMs were stimulated with different doses of GnIH or 100 nmol/L PGE2 for 24 hours (B). Scale bars: 200 μm. n = 3 each group. (***P < 0.001).

(C) CCK8 assay of cellular proliferation using WT mouse BMMs stimulated with different doses of GnIH and 20 ng/ml M-CSF for 2 days. n = 6 each group.

(D and E) Representative images of BMMs from WT and *Gpr147*$^{-/-}$ mice subjected to the Transwell migration assay and cells were quantified. Scale bars: 200 μm. n=3 each group.

(F) WT and *Gpr147*$^{-/-}$ BMMs stimulated with 20 ng/ml M-CSF for 48 hours and perform CCK8 assay. n = 3 each group.

(G) RT-qPCR analysis of the expression of *Trap*, *Nfatc1* and *Ctsk* in *Gpr147*$^{-/-}$ and WT osteoclasts. n = 3 each group. (*P < 0.05, **P < 0.01).

(H and I) Representative TRAP staining images of the differentiation of *Gpr147*$^{-/-}$ BMMs into osteoclast cultured with 10 μM GnIH for 5 days. Scale bars: 200 μm. Trap^{+} cell numbers and area were measured. n = 3 each group. (*P < 0.05).

Supplemental Figure 5. GnIH/Gpr147 signaling has minimal impact on bone formation and osteoblast.

(A-H) Representative images of calcein double labeling and Goldner's staining of the spinal trabecular bone of 2-month-old *GnIH*$^{-/-}$ male mice (A and C) or *Gpr147*$^{-/-}$ male mice (E and G), Scale bars: 50 μm. The number of BFR/BS (bone formation rate per bone surface), MAR (mineral apposition rate), N.Ob/B.Pm, Ob.S/BS and OS/BS were caclulated. n = 6 each group. (*P < 0.05).

Supplemental Figure 6. Knocking out *GnIH* does not affect *GnIH*$^{-/-}$ BMMs proliferation and migration.

(A) RT-qPCR analysis of the expression of *GnIH* in different bone tissues. n = 3 each group.

(B and C) Representative images of BMMs from WT and *GnIH*$^{-/-}$ mice subjected to the Transwell migration assay and cells were quantified. Scale bars: 200 μm. n = 3 each group.

(D) WT and *GnIH*$^{-/-}$ BMMs stimulated with 20 ng/ml M-CSF for 48 hours and perform CCK8 assay. n = 3 each group.

(E and F) RT-qPCR analysis of the expression of *Rankl* and *OPG* in WT and *GnIH*$^{-/-}$ or *Gpr147*$^{-/-}$ preosteoblastic cells. n = 3 each group. (**P < 0.01, ***P < 0.001)

(G and H) Representative TRAP staining images of the differentiation of BMMs into

osteoclasts cultured with WT or *Gpr147*$^{-/-}$ osteoblasts. Scale bars: 200 μm. Trap^{+} cell numbers and area were measured. n= 3 each group. (***P < 0.001).

Supplemental Figure 7. GnIH treatment of osteoclast precursor cells inhibits Nfatc1 nuclear translocation.

(A and B) Representative TRAP staining images of the differentiation of *Gpr147*$^{-/-}$ BMMs into osteoclast cultured with 3-MA for 5 days. n = 3 each group. Scale bars: 200 μm. Trap^{+} cell numbers and area were measured. (***P < 0.001).

(C and D) The quantification of the percentage of cells with nuclear Nfatc1 in osteoclast precursors treated with10 μM GnIH for 48 hours. n = 3 each group. Scale bars: 200 μm. (**P < 0.01).

(E) The quantification of the percentage of cells with nuclear Nfatc1 in osteoclast precursors treated with Omipalisib for 3 days were measured by immunostaining. n = 3 each group. (***P < 0.001).

(F and G) The quantification of the percentage of cells with nuclear Nfatc1 in osteoclast precursors treated with 3-MA for 3 days were measured by immunostaining. n= 3 each group. Scale bars: 200 μm. (***P < 0.001).

Supplemental Figure 8. The treatment of GnIH rescues bone loss and inhibits osteoclast activity.

(A) Quantitation of femur trabecular bone parameters after 30 days of GnIH treatment in 18-month-old male mice. n = 6 each group. (*P < 0.05).

(B) HE staining images of different tissues from 18 month old male mice after GnIH treatment. Scale bars: 200 μm, n = 6 each group.

(C and D) Representative TRAP staining images of the differentiation of $GnIH^{-/-}$ BMMs into osteoclast cultured with 1 ng/ml IL-1β and 10 μM GnIH for 5 days. $Trap^{+}$ cell numbers and area were measured. Scale bars: 200 μm. n = 3 each group. (*P < 0.05, **P < 0.01, ***P < 0.001).

(E and F) Quantitation of femur trabecular bone parameters from Sham, OVX or 0.1 mg/kg GnIH treatment OVX mice for 30 days. Representative uterus images of Sham, OVX or 0.1 mg/kg GnIH treatment OVX mice for 30 days. Scale bars: 5 mm. (*P < 0.05, **P < 0.01).

Supplemental Figure 9. GnIH treatment alleviate LPS-induced osteoporosis.

(A) Quantitation of femur trabecular bone parameters from 0.1 mg/kg GnIH treatment LPS-induced male mice for 7 days. (*P < 0.05, **P < 0.01).

(B and C) Representative micro-CT images of femurs (top; scale bars:1 mm) and trabecular bone (bottom; scale bars: 500 μm) from 0.1 mg/kg GnIH treatment LPS-induced female (B) mice for 7 days. Quantitation of femur trabecular bone parameters from 0.1 mg/kg GnIH treatment LPS-induced female mice for 7 days. n =

4 each group. (*P < 0.05, **P < 0.01).

(D and E) Representative TRAP staining images of the differentiation of BMMs into osteoclast cultured with 50 ng/ml LPS and 10 μM GnIH for 5 days. Scale bars: 200 μm. $Trap^+$ cell numbers and area were measured. n =3 each group. (*P < 0.05, **P < 0.01).

(F) After administering 0.1 mg/kg of GnIH treatment to male mice induced with LPS for 7 days. Serum TNF-α, IL-1β and IL-6 content was detected by ELISA. n = 6 each group. (**P < 0.01, ***P < 0.001).

Supplemental Figure 10. Green light therapy prevents bone loss in OVX mice.

(A) Quantitation of femur trabecular bone parameters. n = 6 each group. (**P < 0.01).

Supplemental Figure 11. Green light therapyt can only partially improve the bone parameters of *GnIH*$^{-/-}$ and *Gpr147*$^{-/-}$ mice.

(A) *GnIH*$^{-/-}$ and *Gpr147*$^{-/-}$mice were exposed to green (520-525 nm, 400 Lux) light therapy for 8 hours (8:00 am-16:00 pm) for 60 days.

(B-E) Representative micro-CT images of femur (top; scale bars: 1 mm) and trabecular bone of the (bottom; scale bars: 500 μm) from *GnIH*$^{-/-}$ or *Gpr147*$^{-/-}$male mice with or without green light exposure. Quantitation of femur trabecular bone

parameters from *GnIH*$^{-/-}$ or *Gpr147*$^{-/-}$ male mice with or without green light exposure. (*P < 0.05, **P < 0.01).

Supplemental Figure 12. Regular light had little effect on serum GnIH, OCN, TRACP and CTX.

(A-D) Human serum GnIH, bone formation markers (OCN), bone resorption markers (TRACP and CTX) content were detected by ELISA. n = 5. Paired t-test.
