# Supplementary material for: GnIH secreted by green light exposure, regulates bone mass through the activation of Gpr147
Source: Bone Res. 2025 Jan 21;13:13. doi: 10.1038/s41413-024-00389-7 (PMC11751147; doi:10.1038/s41413-024-00389-7)
Supplement: Supplementary file 2 — Supplemental Figure 1-12 [file 41413_2024_389_MOESM2_ESM.pdf]

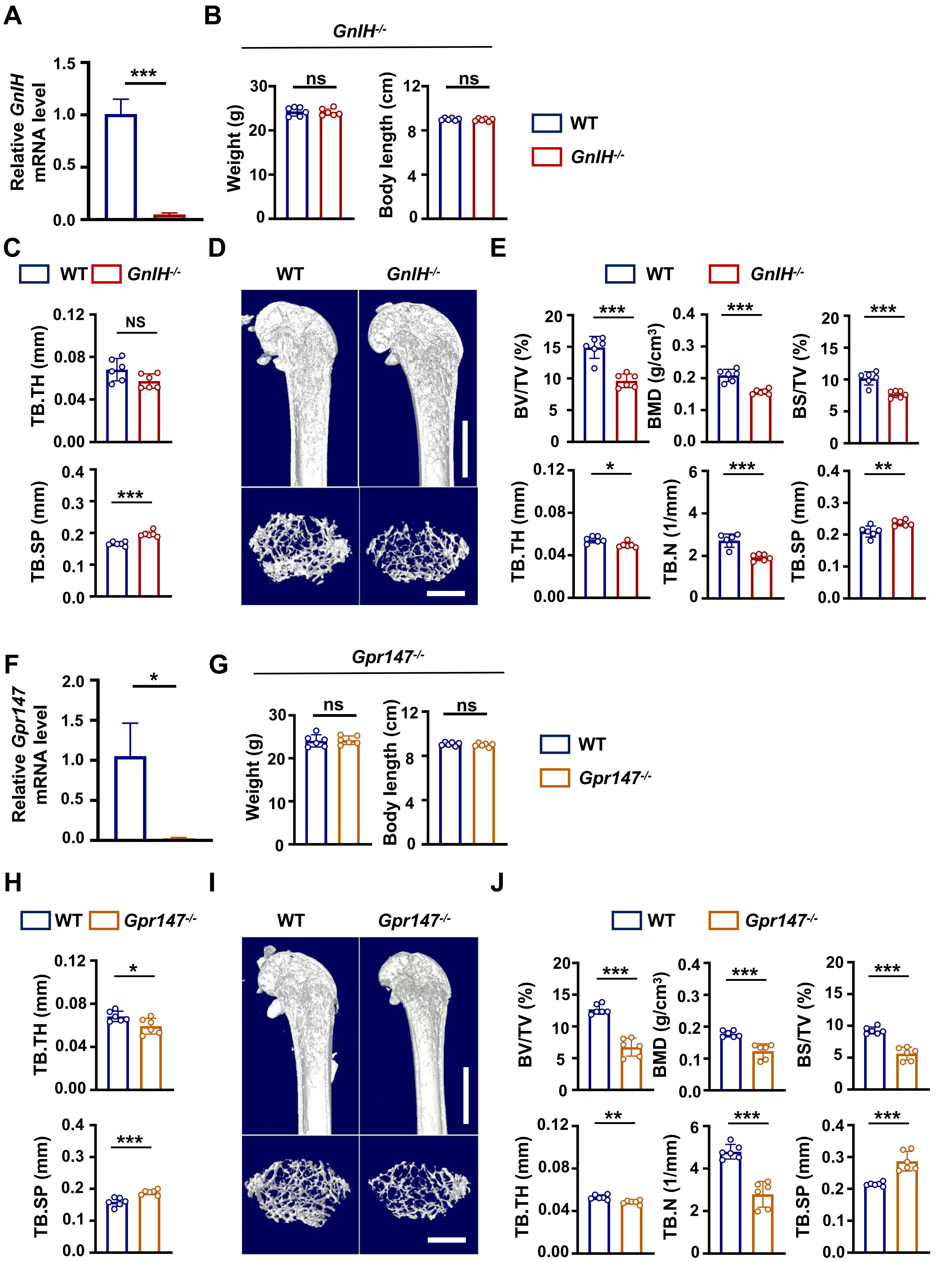
A
Relative GnIH mRNA level
***
B
GnIH-/-
Weight (g)
Body length (cm)
ns
WT
GnIH-/-
C
TB.TH (mm)
NS
TB.SP (mm)
D
E
BV/TV (%)
BMD (g/cm3)
BS/TV (%)
TB.N (1/mm)
F
Relative Gpr147 mRNA level
G
Gpr147-/-
H
I
J

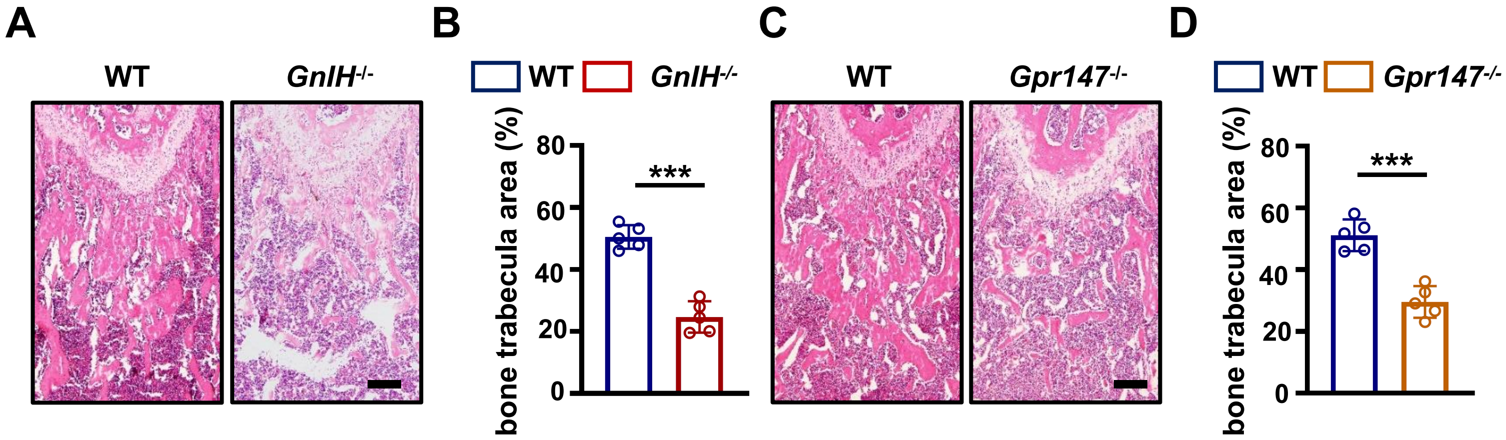
A
WT
GnIH-/-
B
WT
GnIH-/-
bone trabecula area (%)
80
60
40
20
0
***
C
WT
Gpr147-/-
D
WT
Gpr147-/-
bone trabecula area (%)
80
60
40
20
0
***

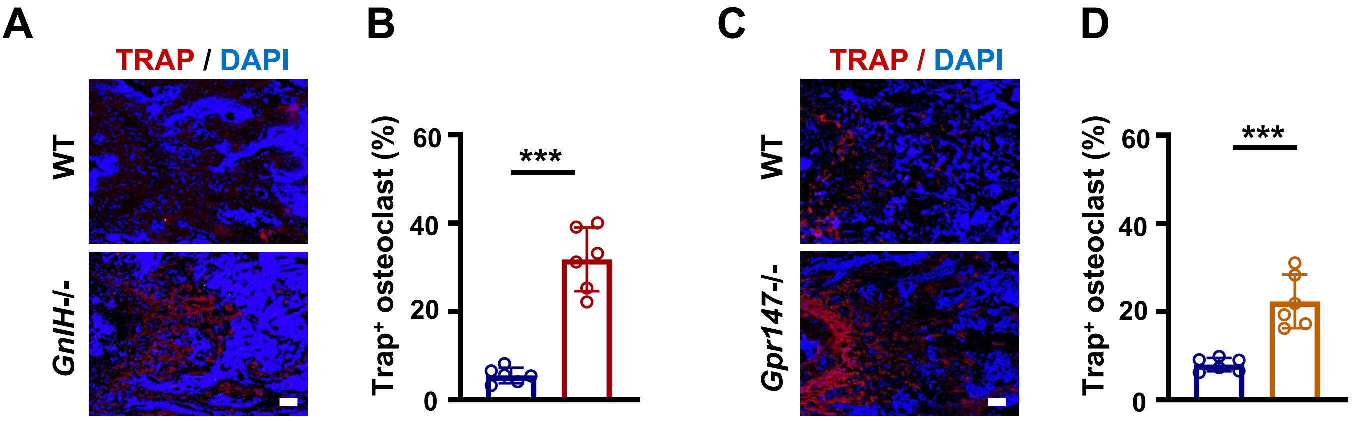
A
TRAP / DAPI
WT
Gnlh-/-
B
Trap⁺ osteoclast (%)
***
0
20
40
60
C
TRAP / DAPI
WT
Gpr147-/-
D
Trap⁺ osteoclast (%)
***
0
20
40
60

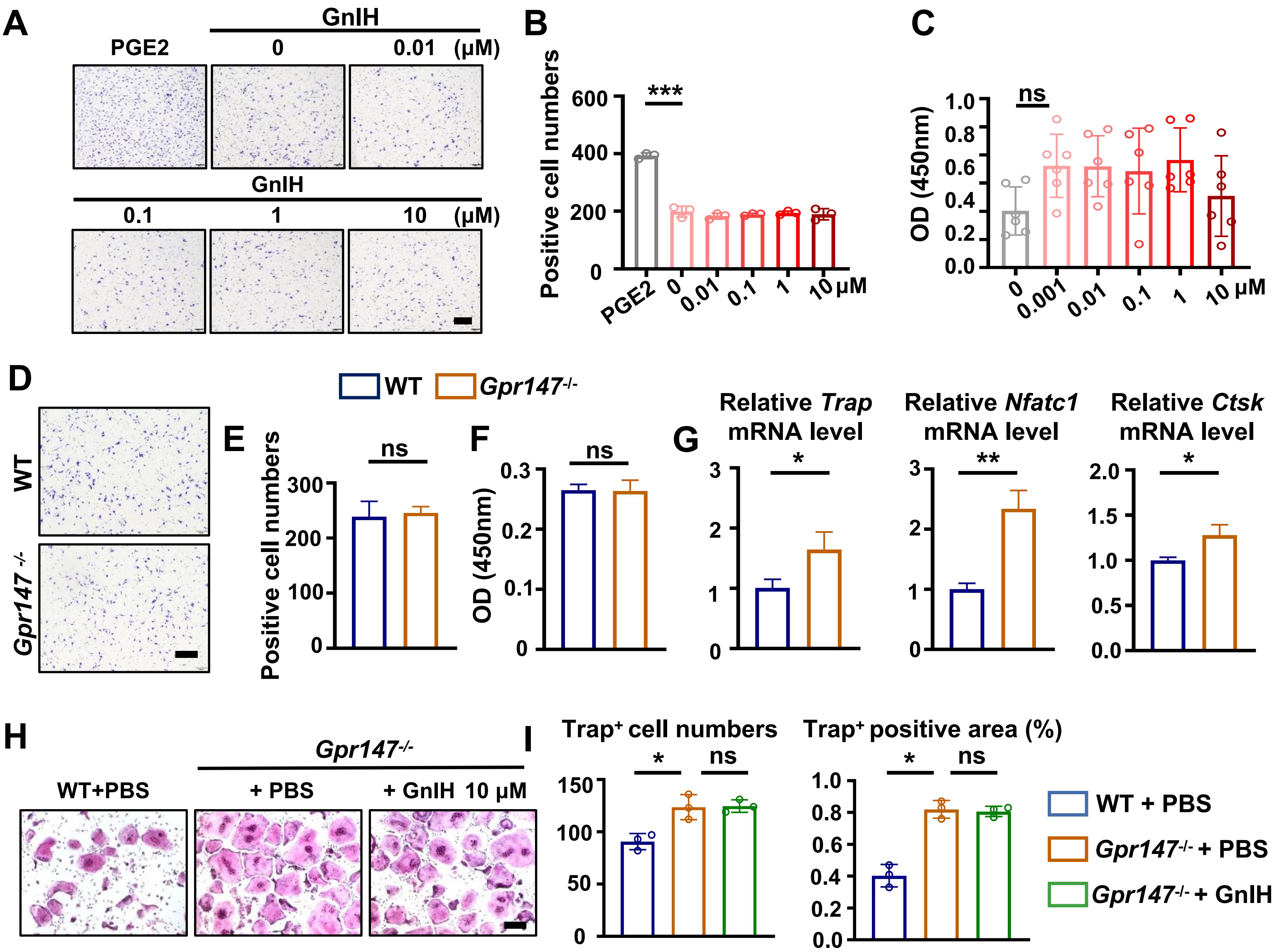
A
PGE2
GnIH
0
0.01 (μM)
0.1
1
10 (μM)
B
Positive cell numbers
***
PGE2
0
0.01
0.1
1
10 μM
C
OD (450nm)
ns
0
0.001
0.01
0.1
1
10 μM
D
WT
Gpr147 -/-
WT
Gpr147-/-
E
Positive cell numbers
ns
F
OD (450nm)
ns
G
Relative Trap mRNA level
*
Relative Nfatc1 mRNA level
**
Relative Ctsk mRNA level
*
H
Gpr147-/-
WT+PBS
+ PBS
+ GnIH 10 μM
I
Trap+ cell numbers
*
ns
Trap+ positive area (%)
*
ns
WT + PBS
Gpr147-/- + PBS
Gpr147-/- + GnIH

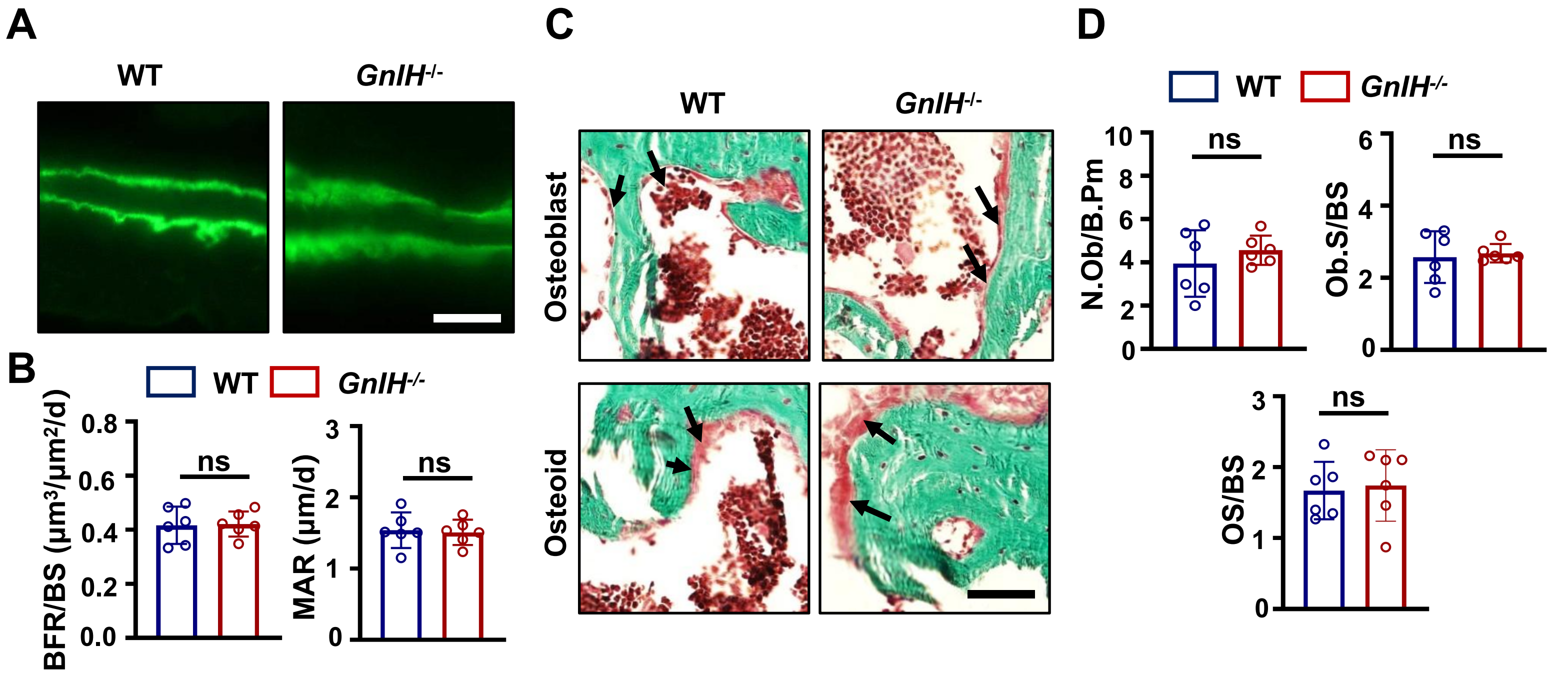
A
WT
GnIH-/-
B
WT
GnIH-/-
BFR/BS (µm³/µm²/d)
MAR (µm/d)
ns
C
WT
GnIH-/-
Osteoblast
Osteoid
D
WT
GnIH-/-
N.Ob/B.Pm
Ob.S/BS
OS/BS
ns

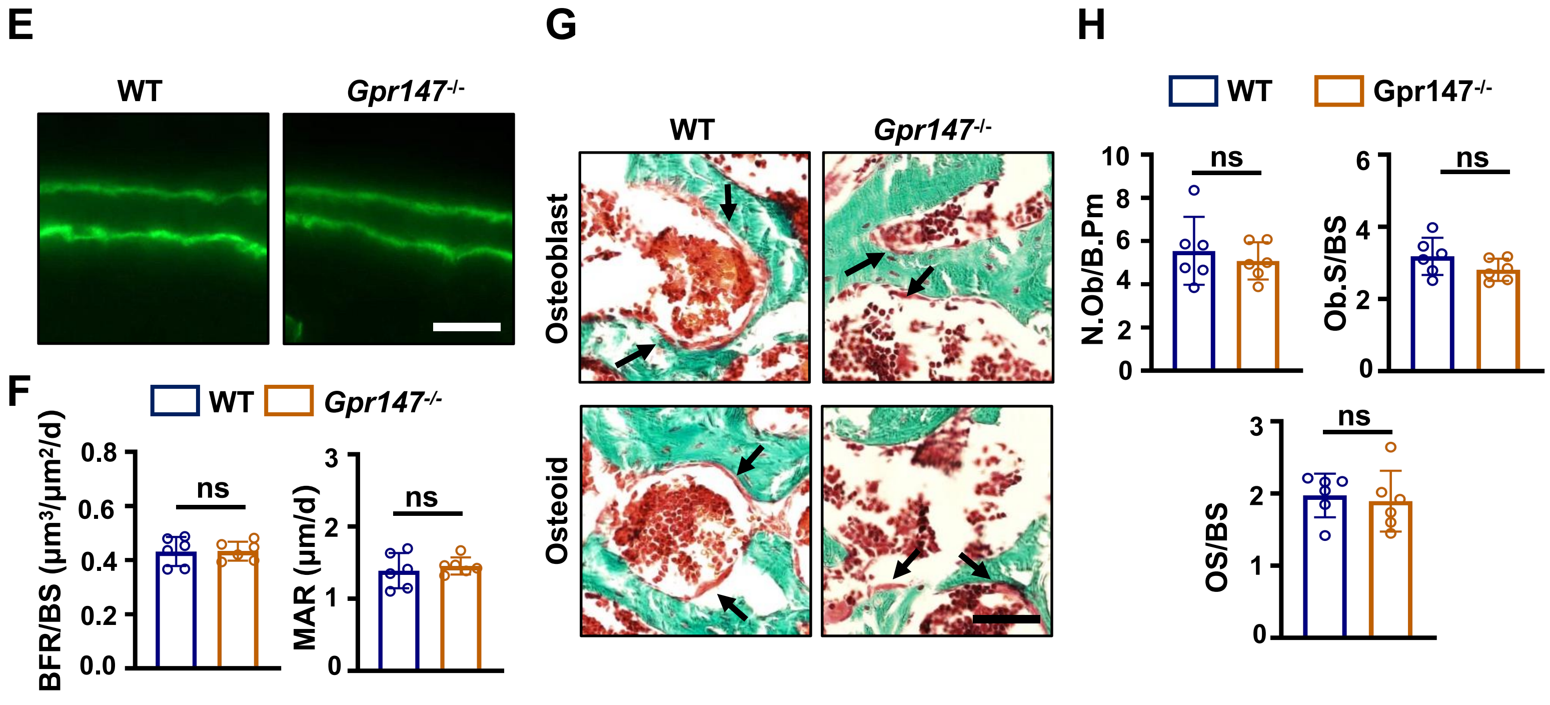
E
WT
Gpr147-/-
F
WT
Gpr147-/-
BFR/BS (µm³/µm²/d)
MAR (µm/d)
ns
G
WT
Gpr147-/-
Osteoblast
Osteoid
H
WT
Gpr147-/-
N.Ob/B.Pm
Ob.S/BS
OS/BS
ns

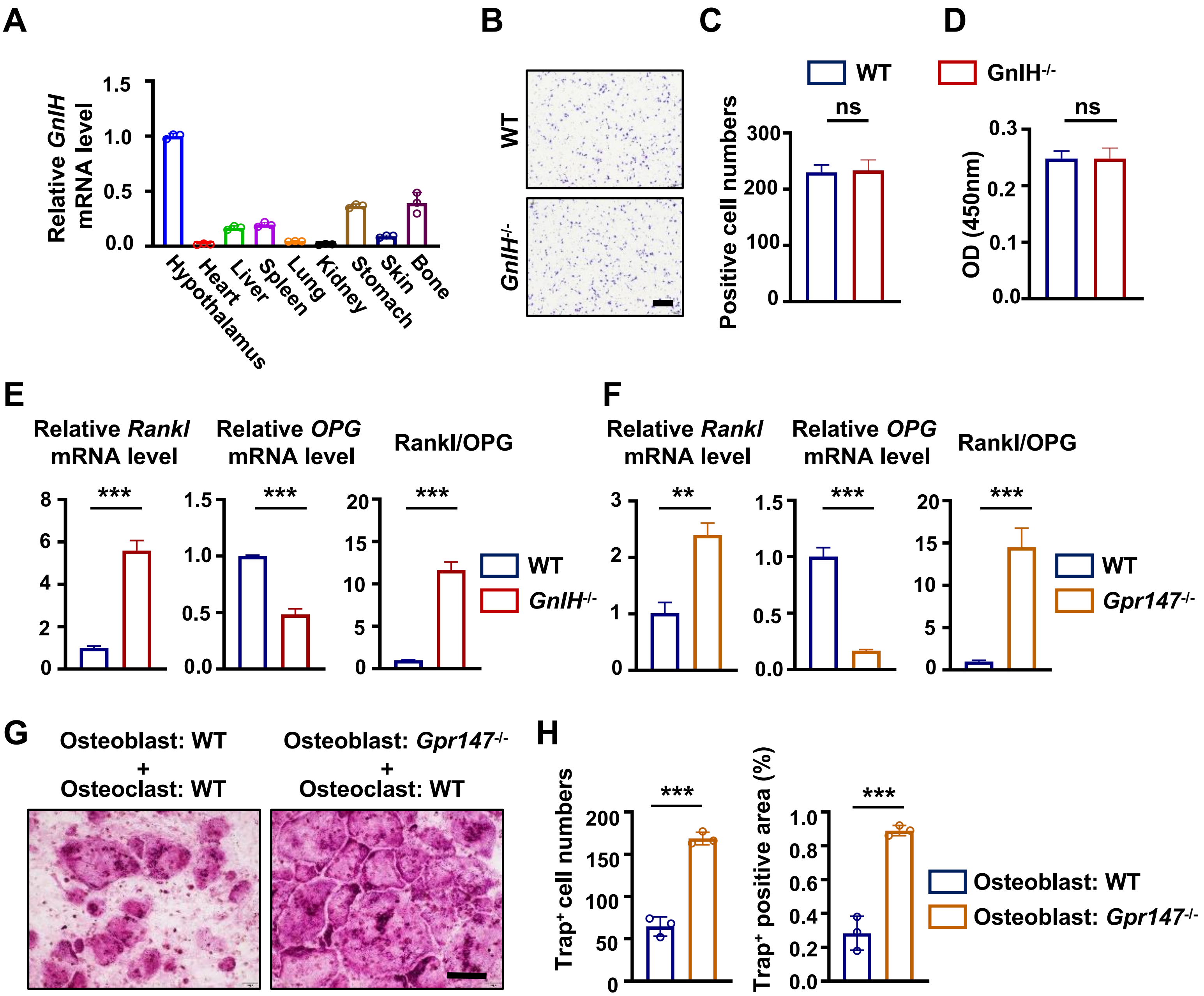
A
Relative GnIH mRNA level
1.5
1.0
0.5
0.0
Hypothalamus
Heart
Liver
Spleen
Lung
Kidney
Stomach
Skin
Bone
B
WT
GnIH-/-
C
WT
GnIH-/-
ns
Positive cell numbers
300
200
100
0
D
ns
OD (450nm)
0.3
0.2
0.1
0.0
E
Relative Rankl mRNA level
Relative OPG mRNA level
Rankl/OPG
***
WT
GnIH-/-
F
Relative Rankl mRNA level
Relative OPG mRNA level
Rankl/OPG
**
***
WT
Gpr147-/-
G
Osteoblast: WT + Osteoclast: WT
Osteoblast: Gpr147-/- + Osteoclast: WT
H
Trap+ cell numbers
Trap+ positive area (%)
***
Osteoblast: WT
Osteoblast: Gpr147-/-

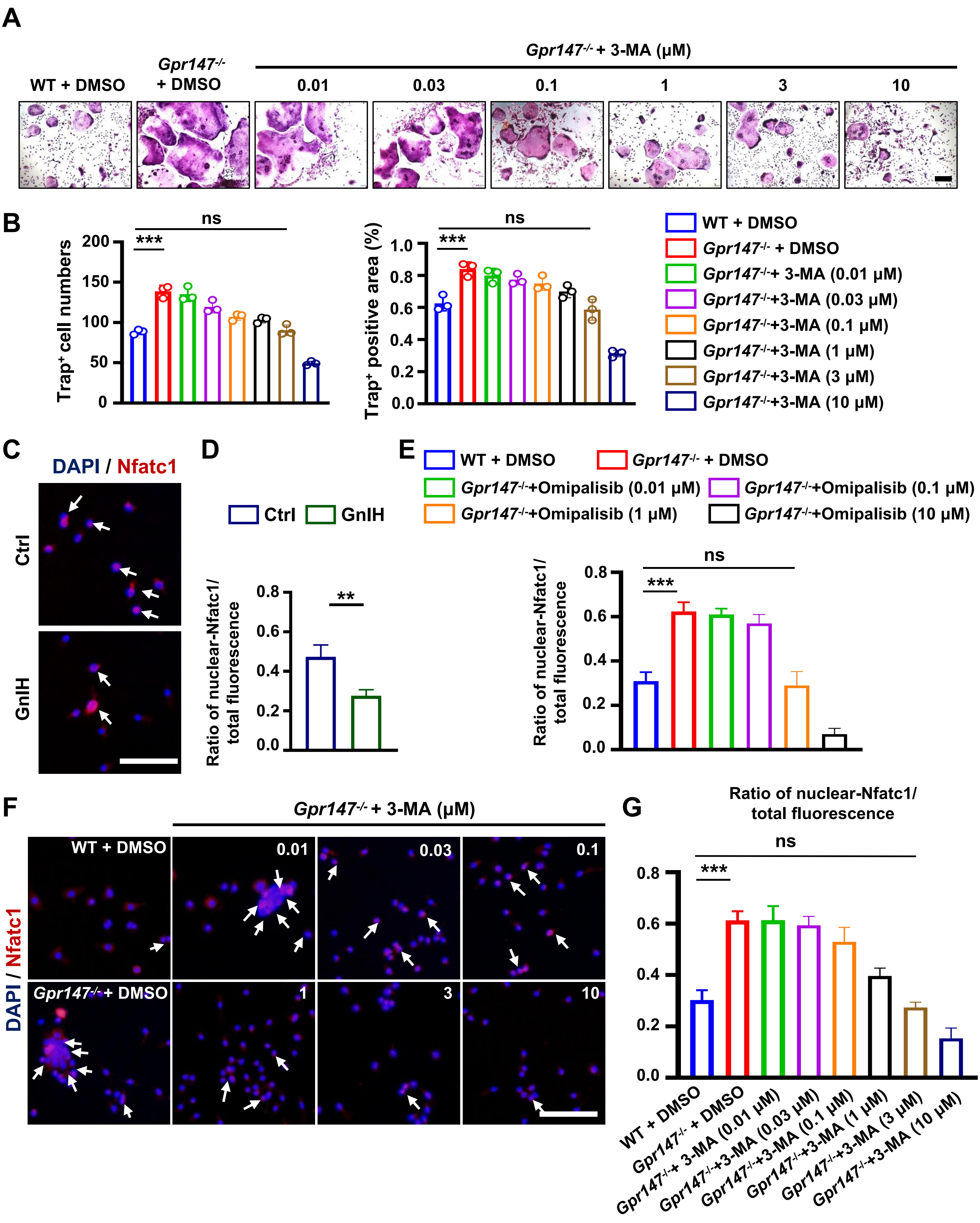
A
WT + DMSO
Gpr147-/- + DMSO
Gpr147-/- + 3-MA (μM)
0.01
0.03
0.1
1
3
10
B
Trap+ cell numbers
Trap+ positive area (%)
ns
***
WT + DMSO
Gpr147-/- + DMSO
Gpr147-/-+ 3-MA (0.01 μM)
Gpr147-/-+3-MA (0.03 μM)
Gpr147-/-+3-MA (0.1 μM)
Gpr147-/-+3-MA (1 μM)
Gpr147-/-+3-MA (3 μM)
Gpr147-/-+3-MA (10 μM)
C
DAPI / Nfatc1
Ctrl
GnIH
D
Ctrl
GnIH
Ratio of nuclear-Nfatc1/ total fluorescence
**
E
WT + DMSO
Gpr147-/- + DMSO
Gpr147-/-+Omipalisib (0.01 μM)
Gpr147-/-+Omipalisib (0.1 μM)
Gpr147-/-+Omipalisib (1 μM)
Gpr147-/-+Omipalisib (10 μM)
Ratio of nuclear-Nfatc1/ total fluorescence
ns
***
F
Gpr147-/- + 3-MA (μM)
WT + DMSO
Gpr147-/- + DMSO
DAPI / Nfatc1
0.01
0.03
0.1
1
3
10
G
Ratio of nuclear-Nfatc1/ total fluorescence
ns
***
WT + DMSO
Gpr147-/- + DMSO
Gpr147-/-+ 3-MA (0.01 μM)
Gpr147-/-+3-MA (0.03 μM)
Gpr147-/-+3-MA (0.1 μM)
Gpr147-/-+3-MA (1 μM)
Gpr147-/-+3-MA (3 μM)
Gpr147-/-+3-MA (10 μM)

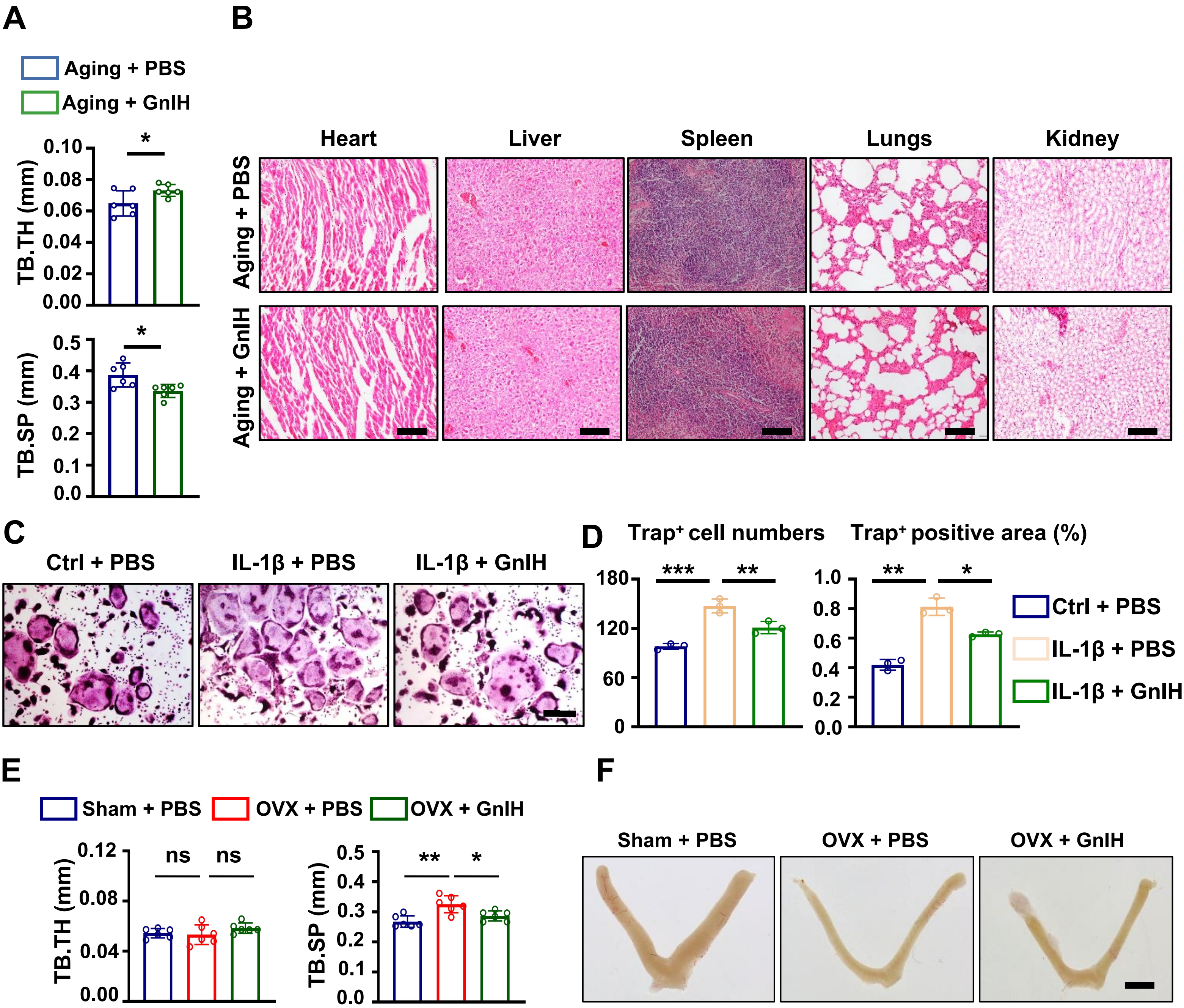
A
Aging + PBS
Aging + GnIH
TB.TH (mm)
0.10
0.08
0.06
0.04
0.02
0.00
*
TB.SP (mm)
0.5
0.4
0.3
0.2
0.1
0.0
*
B
Heart
Liver
Spleen
Lungs
Kidney
Aging + PBS
Aging + GnIH
C
Ctrl + PBS
IL-1β + PBS
IL-1β + GnIH
D
Trap+ cell numbers
Trap+ positive area (%)
180
120
60
0
***
**
1.0
0.8
0.6
0.4
0.2
0.0
**
*
Ctrl + PBS
IL-1β + PBS
IL-1β + GnIH
E
Sham + PBS
OVX + PBS
OVX + GnIH
TB.TH (mm)
0.12
0.08
0.04
0.00
ns
ns
TB.SP (mm)
0.5
0.4
0.3
0.2
0.1
0.0
**
*
F
Sham + PBS
OVX + PBS
OVX + GnIH

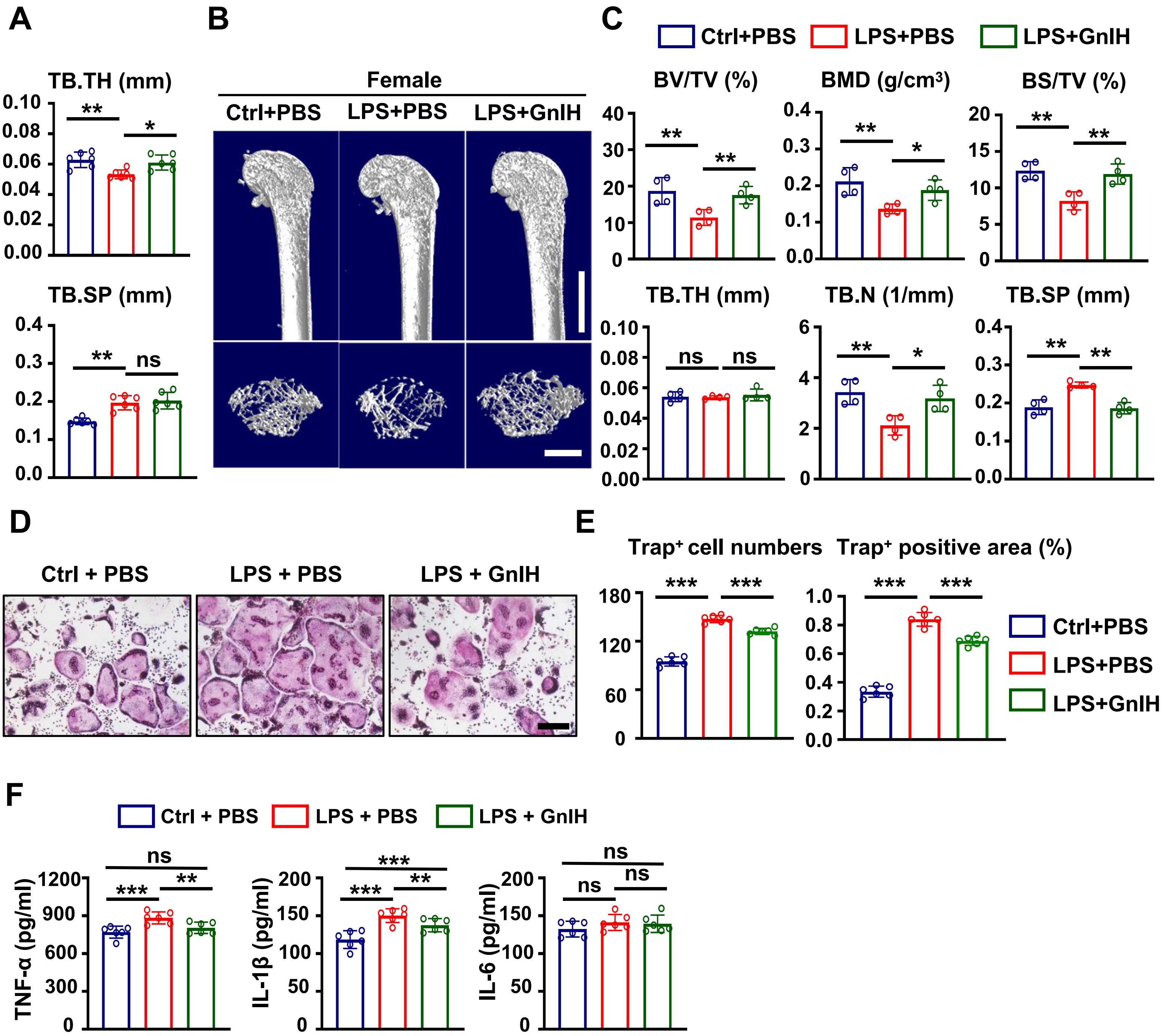
A
TB.TH (mm)
TB.SP (mm)
B
Female
Ctrl+PBS
LPS+PBS
LPS+GnIH
C
Ctrl+PBS
LPS+PBS
LPS+GnIH
BV/TV (%)
BMD (g/cm³)
BS/TV (%)
TB.TH (mm)
TB.N (1/mm)
TB.SP (mm)
D
Ctrl + PBS
LPS + PBS
LPS + GnIH
E
Trap⁺ cell numbers
Trap⁺ positive area (%)
Ctrl+PBS
LPS+PBS
LPS+GnIH
F
Ctrl + PBS
LPS + PBS
LPS + GnIH
TNF-α (pg/ml)
IL-1β (pg/ml)
IL-6 (pg/ml)

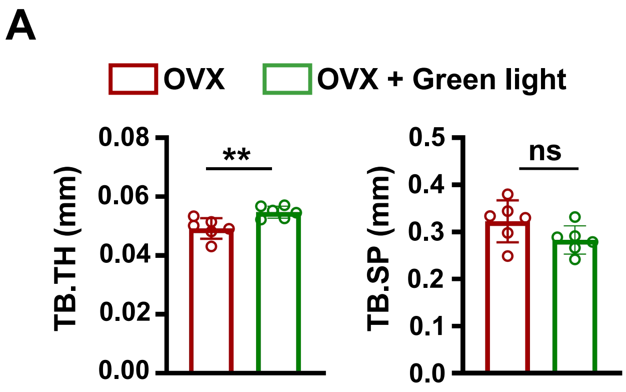
A
OVX
OVX + Green light
TB.TH (mm)
0.08
0.06
0.04
0.02
0.00
**
TB.SP (mm)
0.5
0.4
0.3
0.2
0.1
0.0
ns

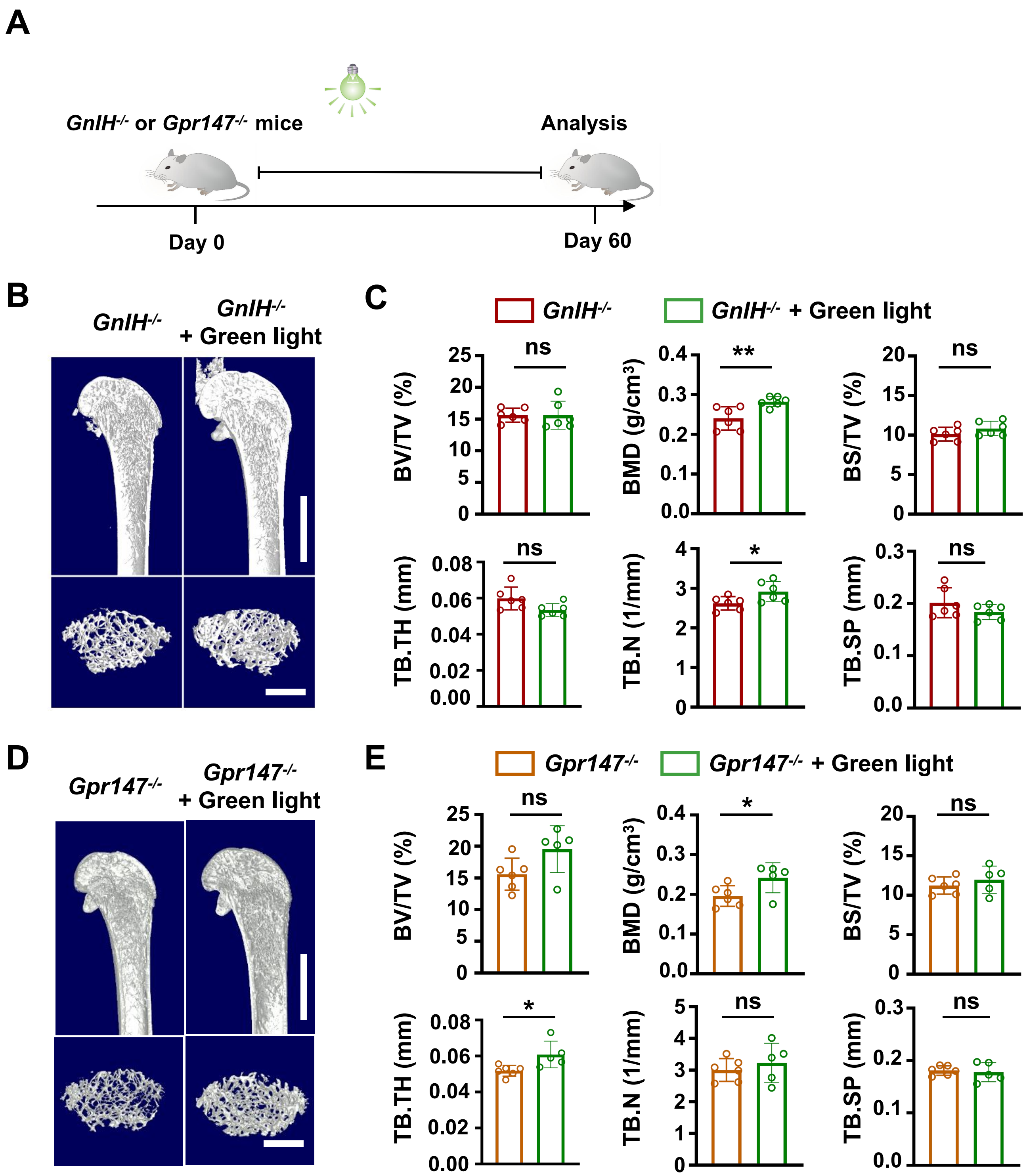
A
GnIH-/- or Gpr147-/- mice
Analysis
Day 0
Day 60
B
GnIH-/-
GnIH-/- + Green light
C
GnIH-/-
GnIH-/- + Green light
BV/TV (%)
BMD (g/cm³)
BS/TV (%)
TB.TH (mm)
TB.N (1/mm)
TB.SP (mm)
ns
**
ns
ns
*
ns
D
Gpr147-/-
Gpr147-/- + Green light
E
Gpr147-/-
Gpr147-/- + Green light
BV/TV (%)
BMD (g/cm³)
BS/TV (%)
TB.TH (mm)
TB.N (1/mm)
TB.SP (mm)
ns
*
ns
*
ns
ns

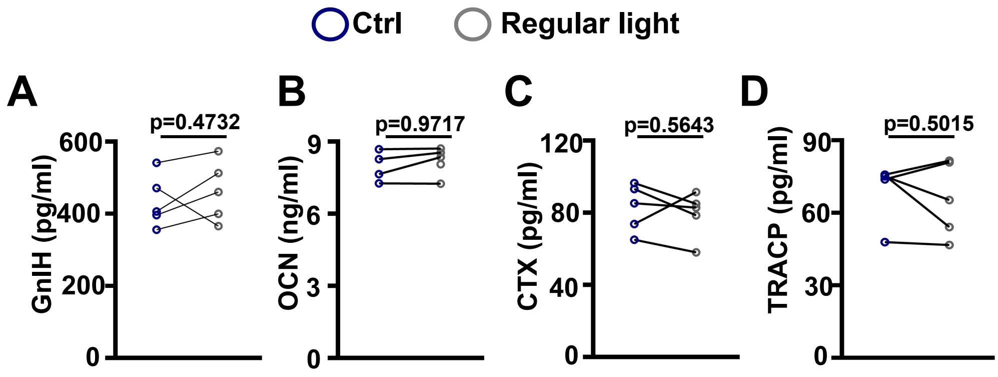
Ctrl
Regular light
A
p=0.4732
GnIH (pg/ml)
600
400
200
0
B
p=0.9717
OCN (ng/ml)
9
6
3
0
C
p=0.5643
CTX (pg/ml)
120
80
40
0
D
p=0.5015
TRACP (pg/ml)
90
60
30
0
